# In Situ Observation of Radiation Damage in Rhabdophane- and Churchite-Type Rare-Earth Phosphate Nanocrystals

**DOI:** 10.1021/acs.inorgchem.6c01299

**Published:** 2026-05-25

**Authors:** Mohamed Ruwaid Rafiuddin, Anamul Haq Mir, Yingjie Zhang

**Affiliations:** † 5419 Australian Nuclear Science and Technology Organisation, Locked Bag 2001, Kirrawee DC, New South Wales 2232, Australia; ‡ Department of Earth Sciences, 67366 University of Cambridge, Downing Site, Cambridge CB2 3EQ, U.K.

## Abstract

Rhabdophane (REPO_4_·H_2_O; RE = La to Dy) and churchite (RE′PO_4_·2H_2_O; RE′ = Gd to Lu and Y) are secondary minerals formed via hydrothermal alteration of primary rare-earth minerals such as monazite (REPO_4_; RE = La to Gd) and xenotime (RE′PO_4_; RE′ = Tb to Lu and Y). Monazite and xenotime are promising host matrices for actinide-rich nuclear wastes. However, the aqueous alteration of these materials could result in the precipitation of rhabdophane and churchite. In this scenario, the actinides could partition into the secondary phases, and the subsequent α-decay of actinides could compromise their structural stability. The effect of radiation on their structure is unknown, and this study determines the radiation stability of rhabdophane ((Sm/Gd/Dy)­PO_4_·H_2_O), churchite ((Gd/Dy/Y)­PO_4_·2H_2_O), monazite (SmPO_4_), and xenotime (DyPO_4_). The materials were irradiated with a 650 keV Xe^2+^ ion beam and the radiation response was monitored in situ using a TEM. Rhabdophane undergoes amorphization at a slightly higher ion fluence than churchite and, therefore, has a relatively higher radiation stability. However, both these materials were outperformed by monazite and xenotime, which demonstrate higher radiation tolerance. This study has demonstrated that rhabdophane and churchite are more susceptible to radiation damage and could have implications on their actinide retention capacity.

## Graphical abstract

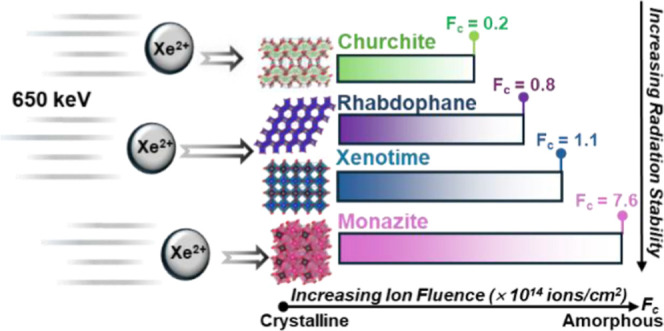

## 1 Introduction

Nuclear energy is a crucial component of the diverse low-carbon energy portfolio.^1^ One of the significant challenge in the nuclear energy sector is the management of radioactive spent nuclear fuel (SNF), which typically contains about ∼96% of uranium (mostly in the form of UO_2_), ∼4% of fission products (e.g., ^131^I, ^137^Cs, ^90^Sr), and long-lived transuranic elements (e.g., ^239^Pu, ^237^Np, ^241^Am).^2^ Many countries consider the SNF as a waste form and opt for its direct disposal in deep geological repositories.^2^ However, some countries reprocess the SNF to recover uranium and plutonium for reuse in the nuclear fuel cycle.^2^ At the reprocessing stage, liquid high-level wastes (HLW) rich in fission products and transuranic elements are produced and must be immobilized in a durable solid waste form before permanent disposal in a geological repository.^2^ While vitrification of the HLW in a glass matrix is the standard method adopted in many countries, significant research over the last few decades has focused on alternate waste forms like ceramic matrices for immobilization of HLW.^3^ A key advantage of ceramic waste forms over glass is their superior radiation stability, chemical durability, and their ability to incorporate specific radionuclides into their crystal lattice.^3^

Synthetic analogues of naturally occurring minerals known to incorporate radionuclides, such as uranium and thorium, are proposed as a ceramic waste form.^4^ Among the various proposed waste forms, monazite (REPO_4_; RE = La to Gd) and xenotime (RE′PO_4_; RE′ = Tb to Lu and Y) are considered as potential host matrices for the immobilization of long-lived radionuclides (e.g., U, Pu, Am, Np, Cm).^5–8^ Both minerals incorporate variable concentrations of Th and U and as a result, have been exposed to displacive radiation damage events over geological time scales.^9–13^ These displacive events, which are primarily initiated by the α-recoil atom produced during the α-decay of Th and U, involve disruptions in the mineral’s atomic arrangement.^4^

As the radiation damage builds up over time, these minerals could eventually become metamict (i.e., radiation-induced transformation from a crystalline to an amorphous state).^14^ However, natural monazite remain predominantly crystalline and are never observed in a fully amorphous state.^9^ This contrasts sharply with synthetic monazite, which become amorphous when exposed to high-energy heavy ion beams (mimicking the α-recoil atom).^9,13,15–17^ This discrepancy has been addressed in the literature and studies suggest that the amorphous domains present in both natural and synthetic monazite are prone to recrystallization through low-temperature thermal annealing and α-particle-induced annealing.^9,16^ While the literature on radiation damage in xenotime minerals is scarce, studies echo the findings for natural monazite, indicating that these minerals are also not found in a fully amorphous state.^18^ Synthetic xenotime are also rendered amorphous by ion-irradiation but can revert to their crystalline state via the same healing mechanism observed in monazite.^19^ The absence of complete metamictization in natural monazite and xenotime is therefore postulated to be the result of this recrystallization of amorphous domains over geological time scales.^9,16,18,19^

The minerals monazite and xenotime are chemically durable as revealed by their resistance to alteration by natural fluids over geological time scales.^20,21^ However, under specific conditions (dictated by the fluid chemistry, pH, and temperature), surface alteration of these minerals can occur during which some of the rare-earths (RE), actinides (Th/U), and P can leach out.^8,20^ In this scenario, the released ions could react and precipitate as a secondary mineral on the surface of the primary mineral via a dissolution-precipitation mechanism; the composition and structure of this mineral could be the same as or different from those of the primary mineral.^20,22–24^ Alternatively, the leached ions could be transported to a new location by the mobile fluid, allowing them to adsorb onto clay/mineral deposits and could react with the local environment therein to precipitate as a secondary mineral.^25–27^

The hydrothermal alteration of monazite and xenotime results in light- and heavy-RE rich alteration fluids, respectively.^27–29^ When these RE-laden fluids—or those derived from other RE-rich sources—combine with the phosphate ions, the resulting precipitation process most commonly yields the secondary hydrated RE phosphates, rhabdophane (REPO_4_·H_2_O; RE = La to Dy) and churchite (RE′PO_4_·2H_2_O; RE′ = Gd to Lu and Y).^26,30–32^ Strikingly, the light- and heavy-RE fractionation (i.e., selective partitioning) in rhabdophane and churchite mirrors that of monazite and xenotime, respectively.^32,33^ It is worth noting that these secondary phases are not exclusively formed by the localized breakdown of monazite and xenotime; they can also precipitate from fluids that have RE from other RE-rich sources (e.g., Bastnäsite—RECO_3_F, ion-adsorption clays etc.), allowing them to exist independent of any specific altered primary mineral.^26,34–36^

Rhabdophane and churchite are supergene minerals (i.e., minerals formed at or near the earth’s surface via alteration of pre-existing minerals) that precipitate in environments characterized by low temperatures and acidic conditions.^32,37^ These minerals exhibit rod-like and acicular (needle-shaped) crystal habits, with rhabdophane generally adopting both morphologies and churchite appearing predominantly acicular.^26,36–38^ Furthermore, their hydrous nature renders these phases metastable; upon heating, rhabdophane and churchite lose their water of hydration and convert to monazite and xenotime, respectively.^39,40^

Like their anhydrous counterparts, these hydrous minerals also incorporate actinides (Th and U) into their crystal structures.^37,41^ In rhabdophane, the Th and U incorporation results in the formation of Th-rich minerals (brockite—(Ca,Th,RE)­PO_4_·H_2_O; grayite—(Th,Pb,Ca)­PO_4_·H_2_O) and U-rich ningyoite ((Ca,U,RE)_2_(PO_4_)_2_·1–2H_2_O) phases, respectively.^41–44^ In contrast, evidence for actinide incorporation in churchite is limited, though few studies confirm the substitution of minor amounts of Th and U within its structure.^37,45^ The capacity of these phases to sequester Th and U therefore underscores their significance in nuclear waste immobilization.^46,47^ In the event of fluid-induced alteration of monazite- and xenotime-based waste forms in a geological repository, actinides may be leached from the host matrix. However, their potential for partitioning into rhabdophane/churchite could significantly limit the transport of actinides into the environment.^23,40^

The role of these secondary phases as actinide hosts is validated by observations from legacy waste sites and laboratory studies; however, these findings are currently limited to rhabdophane, and no analogous findings exist for churchite.^23,48–54^ The Hanford site (USA) is an example of a legacy waste site, containing vast inventories of plutonium (Pu)-containing wastes generated during nuclear weapons production between 1945 and 1987.^49,50^ About ∼200 kg of Pu has been introduced into the environment as a result of disposal of Pu-containing waste into unlined trenches, cribs, and gravel fields.^50^ One of these unlined trenches, the 216-Z-9 trench alone has received about 50–140 kg of Pu.^50^ It was reported that trace quantities of Pu have migrated up to a depth of 37 m below the 216-Z-9 trench.^50^ Consequently, multiple investigations analyzed samples taken from the near- and subsurface sediments beneath this trench to determine the chemical form of Pu.^48,49^ These investigations revealed the presence of Pu-oxide (in the form of PuO_2_ or PuO_2+*x*_) and nanosized Pu–Fe–phosphate phases in the soil; the *d*-spacing values of the latter were consistent with rhabdophane.^48,49^ The identification of this Pu–Fe–phosphate phase represents the first environmental evidence of Pu incorporation into the rhabdophane structure.

Complementing these environmental observations, experimental evidence for Pu-incorporation into the rhabdophane structure was demonstrated through studies detailing the structural and morphological changes in aged (11 to 17 year-old) ^238^Pu-doped monazite single crystals.^51–53^ Single crystals of Eu-monazite doped with ^238^Pu (*t*_1/2_ = 87.7 years) were synthesized and the resulting composition was Eu_0.937_Pu_0.063_PO_4_ (4.9 wt % ^238^Pu + 1.1 wt % of other Pu-isotopes).^51^ Some of these specimens were stored at 20°–25 °C in hermetically sealed glass ampoules filled with air and were monitored over different time intervals.^51^ Surface alteration, characterized by white blisters on the crystal faces, was observed approximately 6 months in storage.^51,52^ After 17 years, some of the faces of the monazite crystal were uniformly coated with a white crust and it was shown that this crust consisted of submicron (Eu, Pu)-rhabdophane precipitates.^53^ These studies provide the first evidence of rhabdophane formation via a nonaqueous pathway, specifically attributing its formation to the combined effects of atmospheric moisture and the recrystallization of radiation damaged zones within the monazite host-matrix.^51,53^

Further evidence of actinide incorporation into rhabdophane stems from leaching experiments conducted on several Th- and U-incorporated phosphate ceramics.^23^ Sintered pellets of phosphate ceramics were exposed to acidic media (0.1 M HNO_3_) for several months, promoting the surface precipitation of the rhabdophane phases (Ca_*x*_Nd_1–2*x*_Th_*x*_PO_4_·1/2H_2_O, Gd-rhabdophane) alongside other secondary phases.^23^ The rhabdophane phase has a low solubility constant and as a result demonstrates superior performance in the long term retention of actinides.^55^ Although no analogous studies have been reported for churchite, it is postulated that this phase will also play a critical role as an actinide solubility-controlling phase. Environmental and experimental observations thus confirm that actinides readily partition into these secondary phases. Consequently, actinide incorporation ensures that these hydrous phases will experience α-decay-induced radiation damage, which influences their structural integrity and long-term actinide retention capacity. Therefore, a comprehensive understanding of the radiation stability of rhabdophane and churchite is critical for predicting the long-term performance of monazite and xenotime host matrices.

To the best of our knowledge, the radiation stability of the rhabdophane and churchite phases has not been investigated, and this study for the first time addresses this critical knowledge gap by investigating the impact of α-recoil atom on the structure of these materials. In this study, high energy heavy ion beams were used to simulate the effects of α-recoil atom. The rhabdophane (REPO_4_·H_2_O; RE = Sm, Gd, Dy) and churchite (RE′PO_4_·2H_2_O; RE′ = Y, Gd, Dy) materials were bombarded with 650 keV Xe^2+^ ions and their structural response was monitored in situ using transmission electron microscopy (TEM). The major focus of this study is to investigate the radiation response of these secondary phases as a function of the RE ionic radii and temperature and compare their structural response to that of the primary actinide host matrices (monazite and xenotime).

## 2 Experimental Section

### 2.1 Synthesis

Materials adopting the rhabdophane- (SmPO_4_·H_2_O, GdPO_4_·H_2_O, DyPO_4_·H_2_O) and churchite-type (GdPO_4_·2H_2_O, DyPO_4_·2H_2_O, YPO_4_·2H_2_O) structures were synthesized using a precipitation method. The following chemicals were used in the synthesis: Sm_2_O_3_ (Alfa Aesar; 99.99%), Gd_2_O_3_ (Alfa Aesar; 99.99%), Dy_2_O_3_ (Alfa Aesar; 99.9%), Y_2_O_3_ (Alfa Aesar; 99.99%), H_3_PO_4_ (Alfa Aesar; 85%), HNO_3_ (Acros-organics; 70%), and NaOH (Fischer Chemicals; AR grade—Pearls). 100 mL of 0.05 M solutions of RE­(NO_3_)_3_ (RE = Y, Gd, Dy) was prepared prior to synthesis by dissolving RE_2_O_3_ (Y_2_O_3_—0.57 g, Gd_2_O_3_—0.91 g, Dy_2_O_3_—0.94 g) in 10 mL of conc. HNO_3_ followed by dilution to 100 mL using a deionized water. Similarly, 1 M H_3_PO_4_ and 8 M NaOH solutions were made prior to synthesis.

#### 2.1.1 Synthesis of Rhabdophane

The Sm-rhabdophane was synthesized by dissolving 0.6775 g of Sm_2_O_3_ in 13.7 mL of conc. H_3_PO_4_ followed by dilution to 100 mL and then refluxing the clear solution at ∼100 °C for 2 h.^56^ The precipitates were washed with deionized water and dried at 60 °C in an oven overnight. For Dy-rhabdophane, 30 mL of 0.05 M Dy­(NO_3_)_3_ was added to a vessel (Savillex PFA) containing 6 mL of 1 M H_3_PO_4_ under continuous stirring and the pH of the clear solution was increased to pH ∼ 1.5 via addition of 8 M NaOH.^57^ The PFA vessel was closed tightly and heated to 45 °C in an oven for 36 h. Gd-rhabdophane was also synthesized using a similar procedure as Dy-rhabdophane but the pH of the solution mixture of Gd­(NO_3_)_3_ and H_3_PO_4_ was increased to 0.95 and heated to 40 °C for 1 day.^57^ Fine precipitates of Gd- and Dy-rhabdophane were washed with deionized water before drying the precipitates at 40 °C in an oven overnight.

#### 2.1.2 Synthesis of Churchite

The Gd-, Dy-, and Y-churchites are synthesized using similar procedure and only differ in the values of pH, reaction temperature, and time duration of the synthesis.^57^ A 30 mL solution of 0.05 M RE­(NO_3_)_3_ (RE = Gd, Dy, Y) was added to a PFA vessel containing 6 mL of 1 M H_3_PO_4_ solution under constant stirring. For Y-churchite, the pH of the solution mixture was adjusted to ∼1.5 and stirred for 1 h and followed by closing the PFA vessel and heating it to 50 °C in an oven for 2 days. In the case of Dy-churchite, the pH of the solution was increased to ∼1.5 and the solution was stirred at room temperature for 3 days prior to heating the PFA vessel containing the solution mixture to 40 °C in an oven for 1 day. For Gd-churchite, the pH of the solution mixture was increased to ∼0.7 and was left stirring for 14 days at room temperature (∼22 °C). All the precipitates were washed with deionized water and dried in air at 40°–50 °C overnight.

### 2.2 Powder X-ray Diffraction (XRD) and Electron Microscopy

Powder XRD patterns of rhabdophane and churchite materials were collected using a Bruker D2 Phaser equipped with a Cu Kα_1,2_ source. The XRD patterns were collected in the 2θ range of 10° to 80° (step size: 0.01°) and the crystal structure of the materials was determined by performing a Rietveld refinement. The refinement was carried out using the Profex (version 4.3.3.) software.^58^ During the refinement, the lattice parameters and the isotropic thermal displacement parameters were allowed to vary while the atomic coordinates and site occupancy factors were kept fixed. The microstructure of these materials was analyzed using scanning electron microscopy (SEM) and TEM. The SEM images of as-synthesized materials was collected in a secondary electron mode at 5 and 15 keV using a TESCAN VEGA instrument. It must be noted that the powders of the as-synthesized materials were not crushed in order to preserve their microstructure. Small quantities of powders of as-synthesized materials were mounted on a carbon tape and coated with a conductive layer of carbon using a Quorum sputter coater. Elemental maps were obtained using energy-dispersive spectroscopy (EDS) using a Bruker FlatQuad detector operated at −20 °C. The EDS spectra and elemental maps were analyzed using the Bruker’s ESPRIT 2.5 software. The TEM images of the as-synthesized materials were collected using a Hitachi H-9500 TEM. The materials were first dispersed in ethanol before placing a drop of the solution on a TEM grid (Holey carbon film supported Ni grids −200 mesh). Using a 300 kV electron beam, bright field TEM (BF-TEM) images and selected area diffraction patterns (SAED) were collected. The TEM images and SAED patterns were processed and analyzed using FIJI software.^59^

### 2.3 In Situ Ion Irradiation

The in situ ion irradiation experiments were carried out at the Microscopes and Ion Accelerators for Materials Investigation (MIAMI-2) facility at the University of Huddersfield, UK. At MIAMI-2, the ion accelerator is coupled to the TEM and allows the observation of radiation-induced structural changes in materials under in situ conditions.^60^ The rhabdophane and churchite materials were irradiated with 650 keV Xe^2+^ ions over a wide temperature range (113 K–773 K) and their structural response was monitored in situ using TEM. For ion-irradiation experiments at cryo- and high-temperatures, the TEM sample grids were mounted on a Gatan liquid nitrogen-cooled holder and a Gatan double tilt heating (DTH) holder, respectively. The irradiation temperature was controlled using Gatan’s SmartSet cold (model 901) and hot (model 900) stage temperature controllers. To ensure uniform cooling or heating, the samples were first equilibrated at the desired temperature for ∼15 min before being exposed to the Xe^2+^ ion beams. Prior to insertion of the sample holder into the TEM column, the current measuring rod/Faraday cup was introduced into the TEM column to measure the incident flux of the Xe^2+^ ion beam (beam spot size ∼ 1 mm). The ion irradiation experiments were carried out over several months, so the values of Xe^2+^ flux (∼1 × 10^12^ to ∼9 × 10^12^ ions cm^–2^ s^–1^) varied slightly between the experiments, though its order of magnitude was kept constant. The ion irradiation was performed in incremental time steps, and this ensured the real-time observation of the radiation-induced structural changes in this material at various ion-fluences (ions/cm^2^). At the time of ion-irradiation, the electron beam was turned off to prevent electron beam induced damage/recrystallization of the samples. Following ion-irradiation, the BF-TEM images and SAED patterns were collected from samples exposed to varying ion-fluences. At the end of the experiment, the sample holder was removed and replaced by the faraday cup to measure the Xe^2+^ flux. The final Xe^2+^ flux is determined by taking the average of the Xe^2+^ flux measured before and after the ion irradiation experiment.

To compare the radiation response of materials possessing different crystal structures, the ion fluences were converted to displacements per atom (dpa). The dpa represents the average number of times every atom in the lattice has been displaced off its site during ion irradiation. The Stopping and Range of Ions in Matter (SRIM) software was used to calculate the dpa values corresponding to various Xe^2+^ ion fluences.^61^ The dpa was calculated in full damage cascade mode for a 650 keV Xe^2+^ ion beam (5000 ions) at an 18.7° incident angle relative to sample surface. A displacement energy (*E*_d_) of 40 eV was used for the RE, P, and O atoms. The X-ray density of the samples, derived from the lattice parameters, was used in these calculations. The dpa values reported in this study correspond to the mean dpa obtained from the near surface region (up to ∼30 nm).

## 3 Results and Discussion

### 3.1 Crystal Structure of Rhabdophane

The crystal structure of rhabdophane has been a topic of debate in the literature, leading to the proposal of various structural models, largely due to the difficulty in growing diffraction-quality single crystals.^62^ Consequently, structural models have been proposed from powder XRD results and, more recently, by employing 3D electron diffraction tomography (EDT) on nanocrystals.^39,62–66^ Early structural work was established in the 1950s by R. C. L. Mooney, who used powder XRD to propose a hexagonal crystal structure for REPO_4_·*n*H_2_O (RE = La, Ce, Nd) in which the water molecules were proposed to occupy the open hexagonal channels.^63,64^ According to that study, analysis of powder XRD patterns revealed only one systematic absence of reflections (i.e., (00*l*) peaks are absent unless l = 3*n*), which resulted in the assignment of two possible space groups (*P*3_1_21 or *P*6_2_22).^63,64^ The simulated powder XRD patterns of CePO_4_·*n*H_2_O with the *P*3_1_21 and *P*6_2_22 space groups are shown in Figure S1 in the Supporting Information.^63,64^ It can be seen in Figure S1 that the powder XRD patterns of these two space groups appear identical.

A subsequent structural investigation on bismuth phosphates by Mooney in the 1960s provided crucial indirect evidence for addressing the rhabdophane space group ambiguity.^67^ That study identified three polymorphs of bismuth phosphates, one of which was reported to be isostructural with rhabdophane. Single crystal XRD analysis of this polymorph conclusively established a trigonal space group (*P*3_1_21) for BiPO_4_·*n*H_2_O although the water molecules were not located.^67^ Based on this observation, it was inferred in that study that the rhabdophane likely adopted the trigonal space group; however, it was noted that further investigation was required.^67^ Utilizing this proposed trigonal structure, Romero et al. refined the powder XRD pattern of BiPO_4_·*n*H_2_O, demonstrating an improved fit by including the water oxygen atoms. As a result, a stoichiometry of BiPO_4_·0.667H_2_O was proposed.^68^

Mesbah et al. later determined the rhabdophane structure using high-resolution synchrotron powder XRD data.^65^ It was shown that the Rietveld refinement of XRD data collected on well crystallized Sm-rhabdophane (SmPO_4_·*n*H_2_O) powders using Mooney’s structural model resulted in unsatisfactory fits.^65^ Instead, it was shown that the Sm-rhabdophane crystallizes in a monoclinic crystal system with a final stoichiometry of SmPO_4_·0.667H_2_O.^65^ This stoichiometry is notably similar to the BiPO_4_·0.667H_2_O proposed in the earlier work by Romero et al.^65,68^ Studies utilizing 3D EDT on rhabdophane nanocrystals yielded hexagonal structural models that differed in their space group assignment: Mayence et al. determined a hexagonal (*P*6_2_22) space group for GdPO_4_·*n*H_2_O, while the more recent study by Duran et al. provided definitive evidence for the presence of 3-fold rotation symmetry axis in DyPO_4_·*n*H_2_O and conclusively establishing the trigonal (*P*3_1_21) space group.^62,66^

In the current study, the trigonal (*P*3_1_21) structural model proposed by Duran et al. and Romero et al. has been used to describe the crystal structure of rhabdophane.^62,66^ The structure of DyPO_4_·H_2_O consists of chains of alternating DyO_8_ and PO_4_ polyhedra, connected via edge-sharing (Figure 1a). These chains are connected to one another via edge-sharing of the DyO_8_ polyhedra, resulting in a framework structure containing open channels running parallel to the *c*-axis, in which the water molecules reside (Figure 1a). Compared to the ideal hexagonal channel in Mooney’s initial model, the slight displacement of the cations in this structure distorts the channel’s geometry, causing it to tend toward a triangular pore geometry (Figure 1b,c).^62^ Notably, this feature was also observed in the monoclinic structural model proposed by Mesbah et al.^62,65^ It was mentioned by Duran et al. that the cation displacement occurs within the confines of trigonal symmetry (Figure 1c).^62^

**Figure 1:**
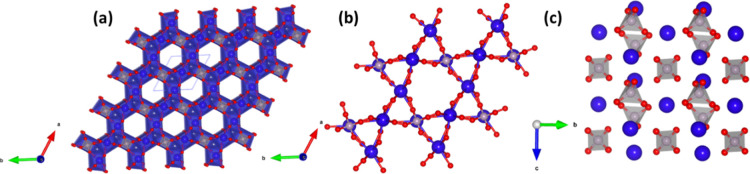
(a) Crystal structure of DyPO_4_·H_2_O. The DyO_8_ polyhedra and PO_4_ polyhedra are shown in blue and gray. (b) Ball and stick representation showing the channel’s triangular pore-like geometry. (c) The displacement of Dy and P cations in the trigonal structure resulting in their zigzag arrangement. The crystal structures were generated using the VESTA software.^69^

The powder XRD patterns of as-synthesized REPO_4_·H_2_O materials (RE = Sm, Gd, Dy) are shown in Figure 2, alongside the reference XRD pattern of the trigonal structural model. The materials are phase-pure and a noticeable shift (Figure 2) in the diffraction peaks to higher 2θ angles was observed with decreasing RE ionic radii (Sm^3+(VIII)^ = 1.079 Å, Gd^3+(VIII)^ = 1.059 Å, Dy^3+(VIII)^ = 1.027 Å). The lattice constants were obtained via Rietveld refinement of their powder XRD patterns (Figure S2 in the Supporting Information) using a modified trigonal structural model (DyPO_4_·H_2_O) in which the structural coordinates of water oxygen atoms from the BiPO_4_·0.667H_2_O was also included.^62,68^ The inclusion of oxygen atoms from water in the structural model resulted in a significant improvement in the fit of the experimental diffraction patterns. The lattice constants of the REPO_4_·H_2_O materials decrease with decreasing RE ionic radii (Table 1). The rhabdophane structure is stable up to 600 °C (873 K) and the powder XRD patterns of the annealed materials are presented in Figure S3 in the Supporting Information. Above 600 °C (873 K), the rhabdophane structure transforms to a stable monazite structure.^56^

**Figure 2:**
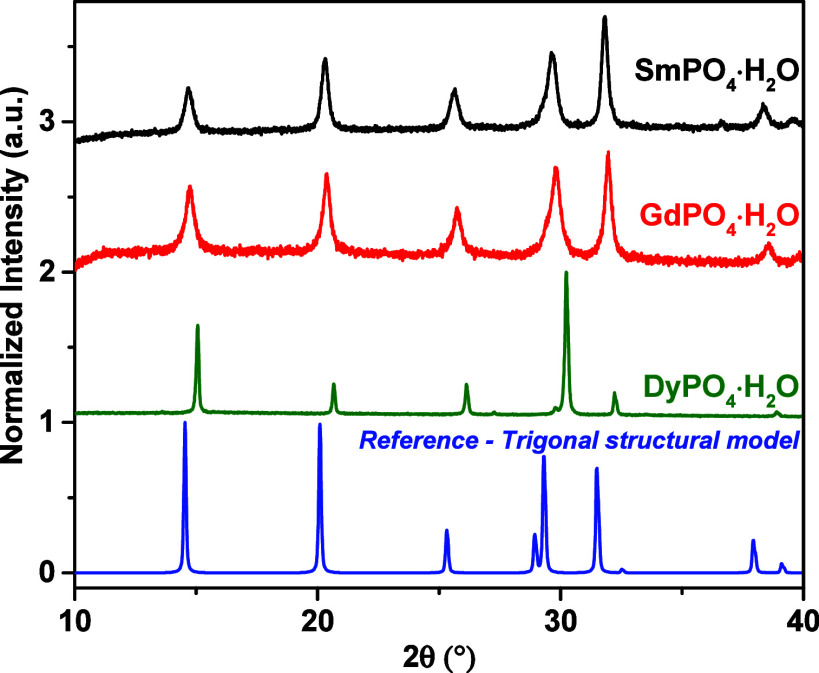
Powder XRD patterns of REPO_4_·H_2_O (RE = Sm, Gd, Dy) presented along with the reference XRD pattern of DyPO_4_·H_2_O.

**Table 1:** Lattice Constants of Rhabdophane (Sm/Gd/Dy)­PO_4_·H_2_O

rhabdophane	*a* (Å)	*c* (Å)	unit cell volume *V* (Å^3^)
SmPO_4_·H_2_O	6.9485 (3)	6.3584 (3)	265.8
GdPO_4_·H_2_O	6.9069 (5)	6.3259 (4)	261.2
DyPO_4_·H_2_O	6.8536 (1)	6.3157 (2)	256.9

### 3.2 Crystal Structure of Churchite

The structure of churchite has been reported in two studies: one by Kohlmann et al. on natural churchite (Y_1–*x*_(Gd,Dy,Er)_*x*_PO_4_·2H_2_O) mineral and another by Ivashkevich et al. on synthetic churchite (YPO_4_·2H_2_O).^70,71^ Both the studies conclude that churchite is isostructural with gypsum (CaSO_4_·2H_2_O) and crystallizes in a monoclinic crystal system (space group: *C*2/*c*).^70,71^ The crystal structure is layered, with sheets composed of YO_8_ and PO_4_ polyhedra linked by edge-sharing (Figure 3). These layers are held together via interlayer hydrogen bonding. In the YO_8_ polyhedra, the Y is coordinated to six oxygen atoms from phosphate anions, while the remaining two oxygen atoms are provided by the water molecules.

**Figure 3:**
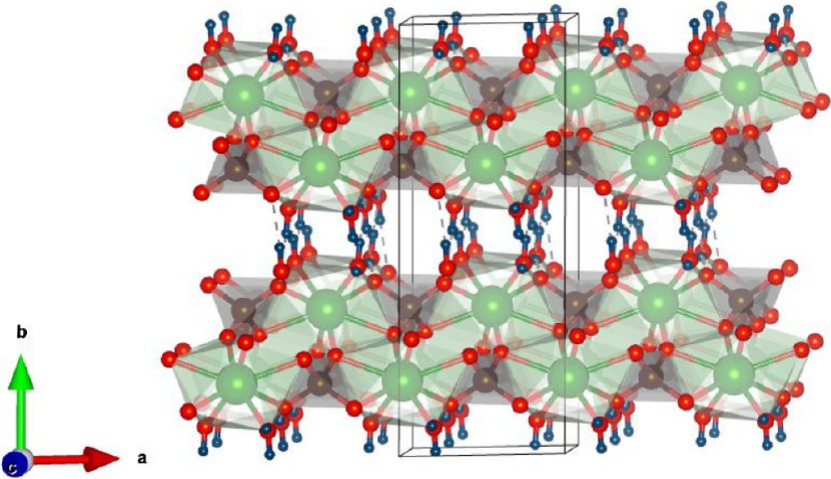
Crystal structure of YPO_4_·2H_2_O. The layers consist of YO_8_ (green) and PO_4_ (gray) polyhedra. The water oxygen atoms are shown in blue, and the layers are held together by the H-bonds shown in dotted lines. The crystal structure was generated using VESTA.^69^

The XRD patterns of RE′PO_4_·2H_2_O (RE′ = Gd, Dy, Y) are presented in Figure 4, alongside the reference pattern of monoclinic YPO_4_·H_2_O. All materials were confirmed to be phase pure. Consistent with the trend observed for the rhabdophane materials, the diffraction peaks systematically shift to higher 2θ values as the RE ionic radii decreases (Gd^3+(VIII)^ = 1.059 Å, Dy^3+(VIII)^ = 1.027 Å, Y^3+(VIII)^ = 1.019 Å). Rietveld refinement of the XRD patterns of RE′PO_4_·2H_2_O was performed using their respective monoclinic structural model (GdPO_4_·2H_2_O, DyPO_4_·2H_2_O, YPO_4_·2H_2_O) to determine the lattice constants (Figure S4 in the Supporting Information).^57,71^ A contraction in the unit cell volume proportional to the decreasing size of rare-earth cation was observed (Table 2). The authors of this study have previously reported that the churchite structure is stable only up to ∼200 °C (473 K) and upon complete removal of water, the structure transforms to a stable xenotime structure.^57^

**Figure 4:**
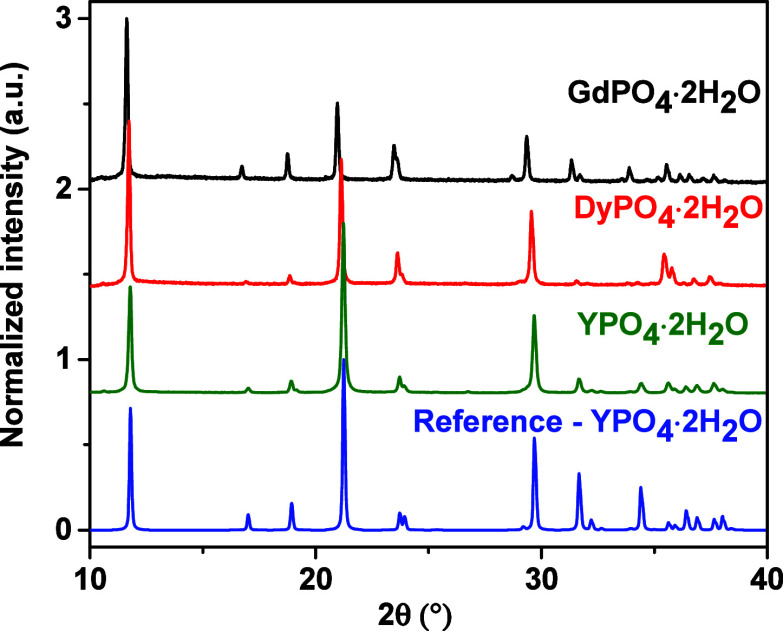
XRD patterns of churchite (RE′PO_4_·2H_2_O; RE′ = Gd, Dy, Y) presented along with the reference XRD pattern from YPO_4_·2H_2_O.

**Table 2:** Lattice Constants of Churchite (Gd/Dy/Y) PO_4_·2H_2_O

churchite	*a* (Å)	*b* (Å)	*c* (Å)	β (deg)	unit cell volume *V* (Å^3^)
GdPO_4_·2H_2_O	6.2157 (1)	15.1259 (3)	5.6222 (1)	114.954 (2)	479.2
DyPO_4_·2H_2_O	6.1747 (5)	15.0453 (4)	5.5969 (2)	115.283 (5)	470.1
YPO_4_·2H_2_O	6.1527 (1)	15.0024 (3)	5.5818 (1)	115.446 (1)	465.2

### 3.3 Crystal Structure of Monazite and Xenotime

The monazite (SmPO_4_) and xenotime (DyPO_4_) materials were obtained via annealing of rhabdophane ((Sm/Dy)­PO_4_·H_2_O) materials to 1000 °C (1273 K) and their crystal structures are presented in Figure 5. Materials adopting the monazite and xenotime structure crystallize in the monoclinic (space group—*P*2_1_/*n*) and tetragonal (space group—*I*4_1_/*amd*) crystal system, respectively.^72^ In SmPO_4_, edge-sharing chains of alternating SmO_9_ and PO_4_ polyhedra are found along the *c* axis and these chains are connected to one another through edge-sharing of SmO_9_ polyhedra (Figure 5a). In SmO_9_ polyhedra, nine unique Sm–O bond distances were observed indicating a distorted polyhedra.^56^ In contrast, the DyO_8_ polyhedra in DyPO_4_ are relatively more symmetrical and contains two sets of unique Dy–O bond distances (Figure 5b).^56^ The DyO_8_ polyhedra are connected to the PO_4_ tetrahedra in a similar fashion as the monazite structure. The powder XRD patterns of monazite and xenotime materials are presented in Figures S5 and S6 in the Supporting Information. The lattice constants obtained via the Rietveld refinement (see Figures S7 and S8 in the Supporting Information) of the powder XRD patterns of monazite and xenotime materials are presented in Table S1 in the Supporting Information.

**Figure 5:**
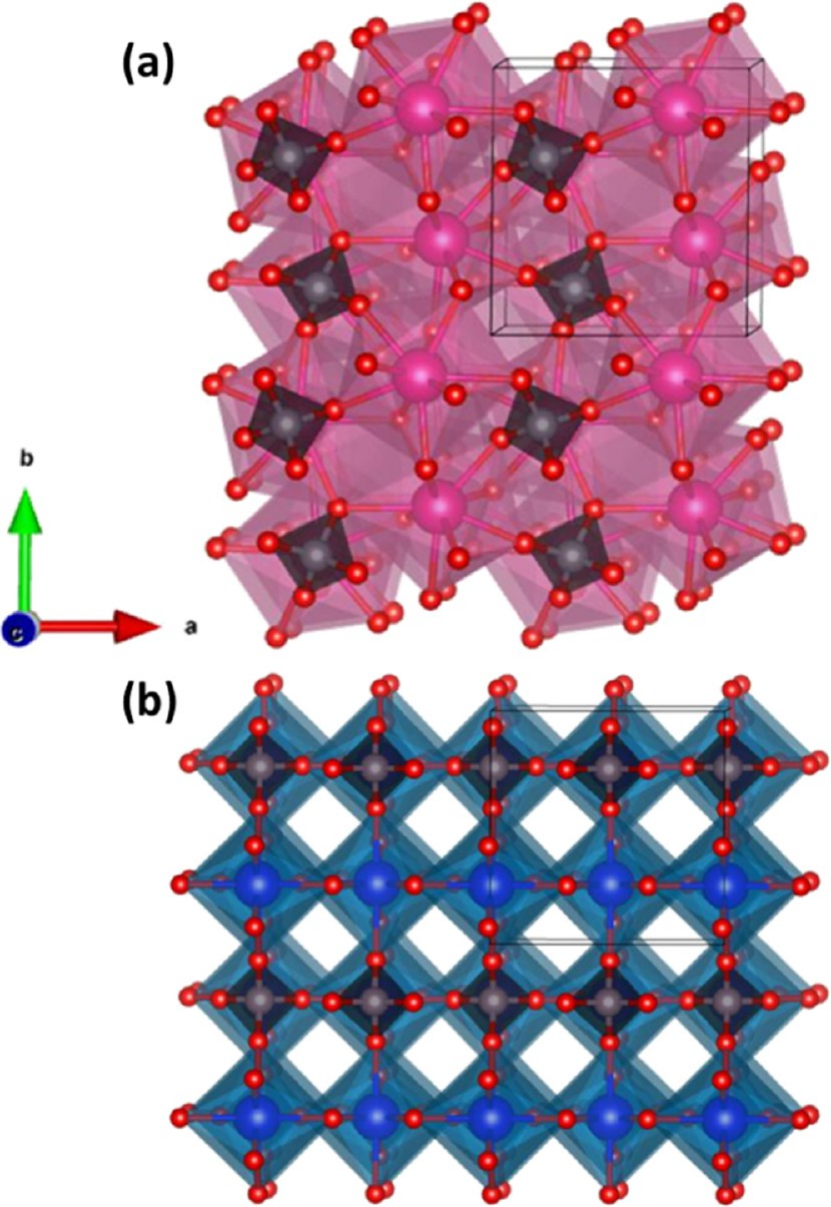
Crystal structure of (a) monazite (SmPO_4_) and (b) xenotime (DyPO_4_). The SmO_9_, DyO_8_, and PO_4_ polyhedra are shown in pink, blue, and black. Crystal structures were generated using VESTA.^69^

### 3.4 Morphological and Microstructural Characterization

#### 3.4.1 Rhabdophane

The high-resolution SEM images of (Sm/Gd/Dy)­PO_4_·H_2_O reveal a distinct morphology that is dependent on both the synthetic conditions and the RE (Figure 6). The Sm-rhabdophane forms dense spherulitic aggregates characterized by radiating rims consisting of rod-like particles (Figure 6).^38^ In contrast, both the Gd- and Dy-rhabdophane exhibit a primary rod-like morphology, with the Dy-rhabdophane rods exhibiting well-defined prismatic facets (Figures 6 and S9 in the Supporting Information). The lack of spherulitic aggregation in Gd- and Dy-rhabdophane is likely a result of differences in their synthesis conditions: the Sm-rhabdophane was synthesized by refluxing a solution of Sm^3+^ and phosphate anions at ∼100 °C, while the Gd- and Dy-rhabdophane were synthesized via the precipitation method at 40° and 45 °C, respectively. Elemental maps acquired via EDS confirm the compositional homogeneity (Figure 7). The EDS spectra of (Sm/Gd/Dy)­PO_4_·H_2_O are presented in Figure S10 in the Supporting Information. TEM analysis revealed the presence of rod-like nano crystals (Figure 8).

**Figure 6:**
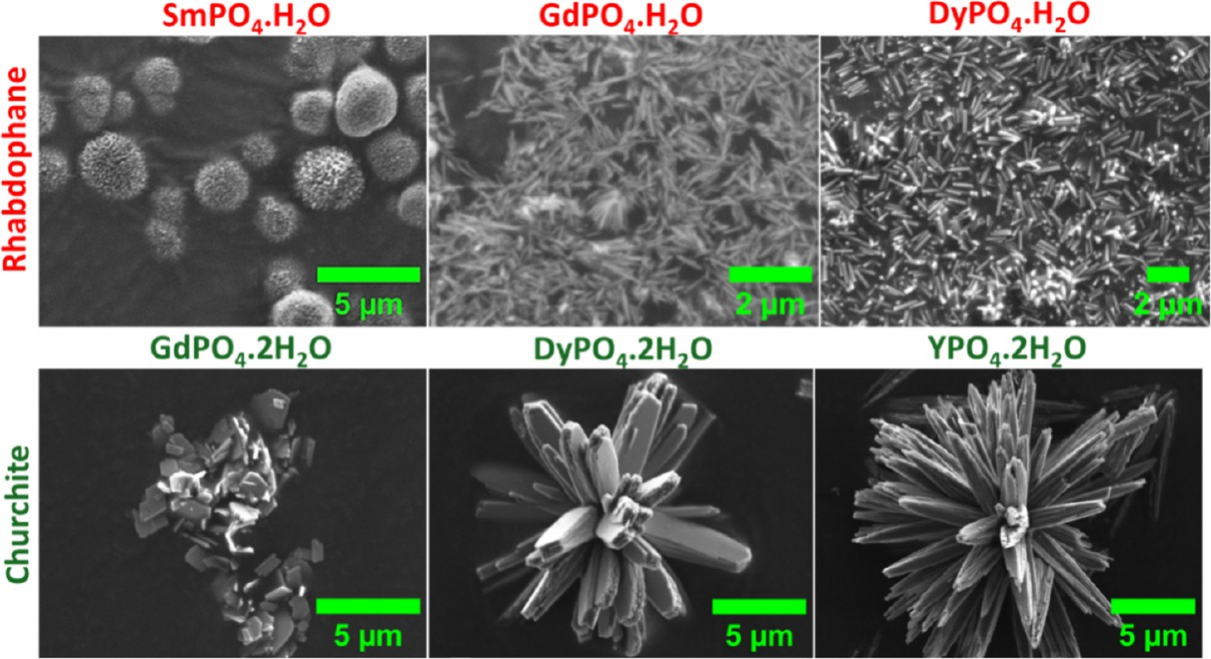
SEM images of rhabdophane ((Sm/Gd/Dy)­PO_4_·H_2_O) and churchite ((Gd/Dy/Y)­PO_4_·2H_2_O).

**Figure 7:**
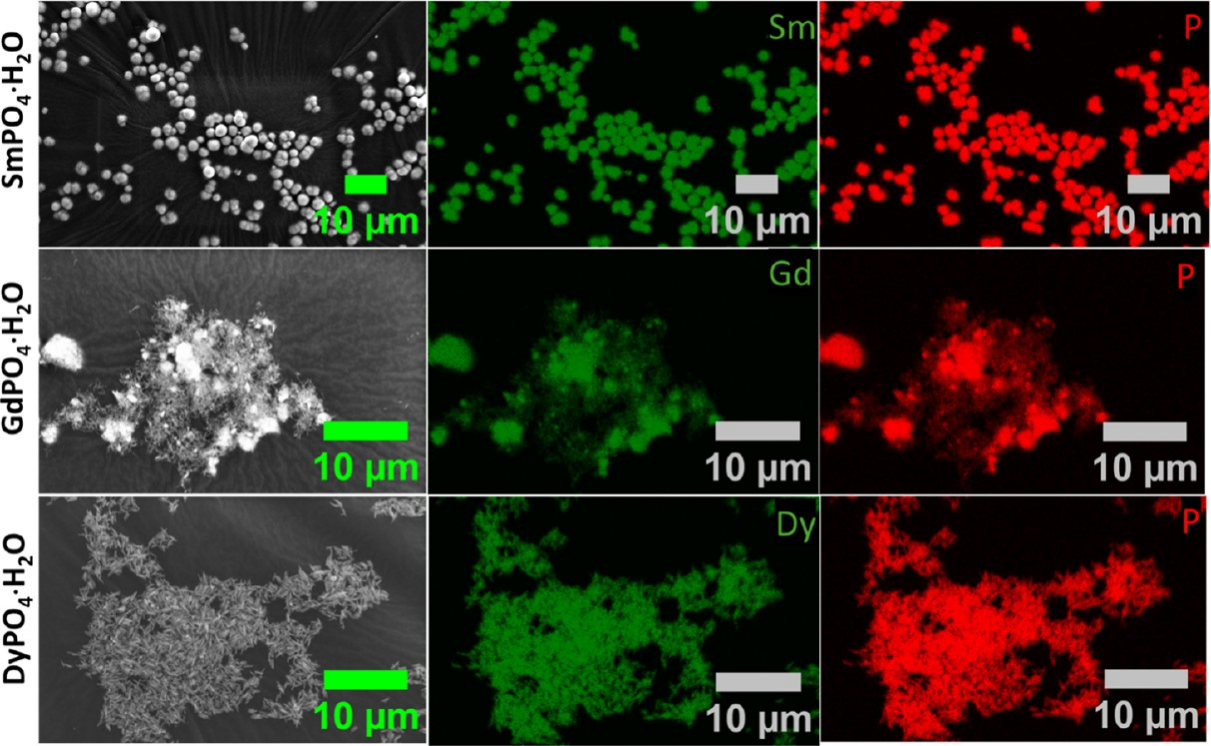
SEM–EDS images of rhabdophane (Sm/Gd/Dy)­PO_4_·H_2_O.

**Figure 8:**
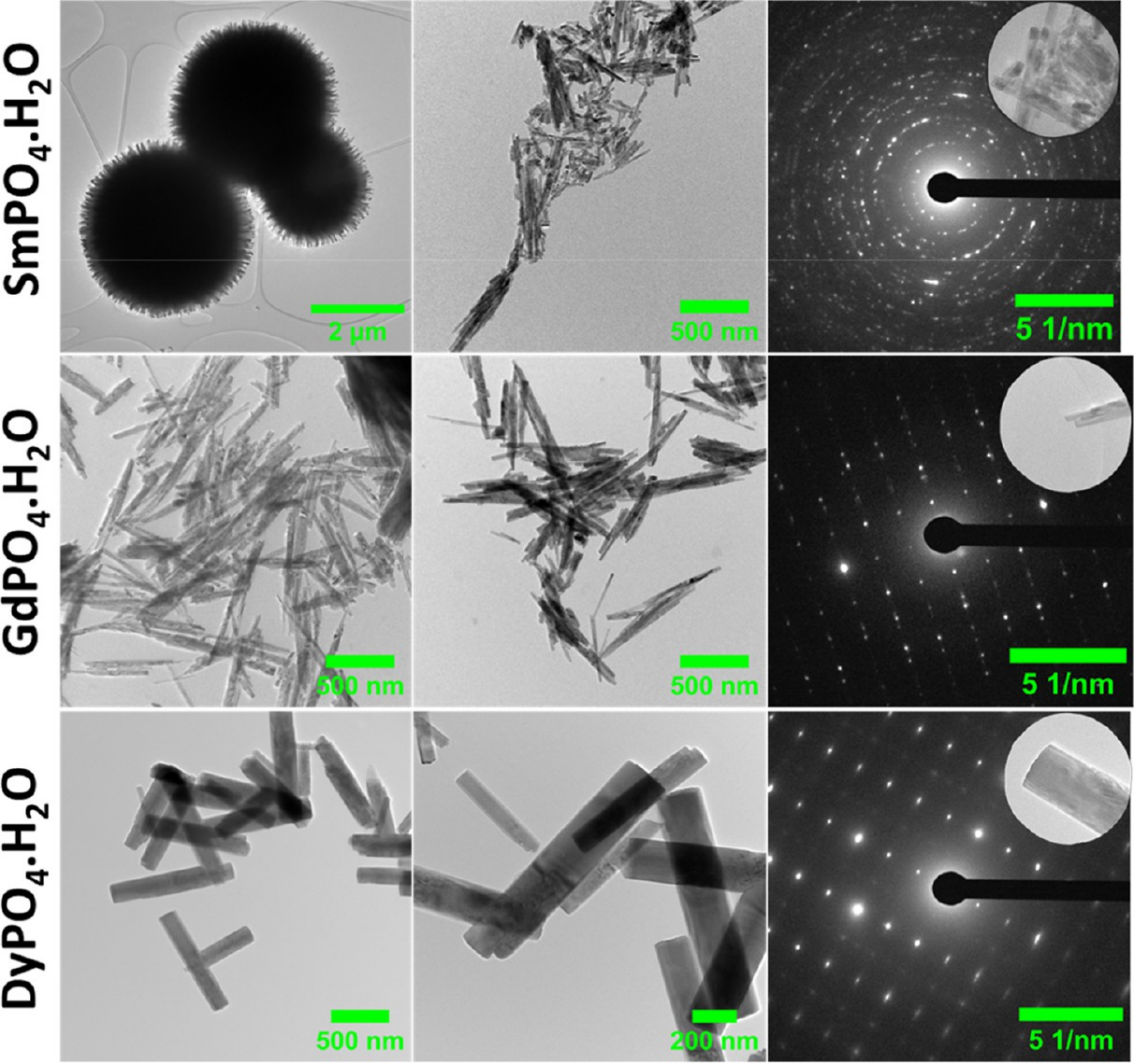
TEM images and SAED patterns of rhabdophane (Sm/Gd/Dy)­PO_4_·H_2_O, exhibiting the presence of rod-like nanocrystals.

#### 3.4.2 Churchite

Mirroring the observations for rhabdophane, the members of the churchite series also exhibit distinct morphologies dictated by synthesis conditions and the RE (Figure 6). The Gd-churchite, synthesized via a room temperature precipitation method, exhibited a plate-like morphology (Figure 6). However, the continuous, accidental stirring of the reaction medium following the initiation of precipitation may have compromised the true primary morphology of the Gd-churchite samples. In contrast, the Dy- and Y-churchite samples consist of aggregates of acicular (needle-like) particles. Note the microstructure was preserved during the synthesis. The elemental maps of (Gd/Dy/Y)­PO_4_·2H_2_O confirm the compositional homogeneity of the RE and P throughout the particles (Figure 9). The EDS spectra of (Gd/Dy/Y)­PO_4_·2H_2_O are presented in Figure S11 in the Supporting Information. TEM analysis further demonstrated that all members of the churchite series are composed of needle-like nano crystals (Figure 10).^57^

**Figure 9:**
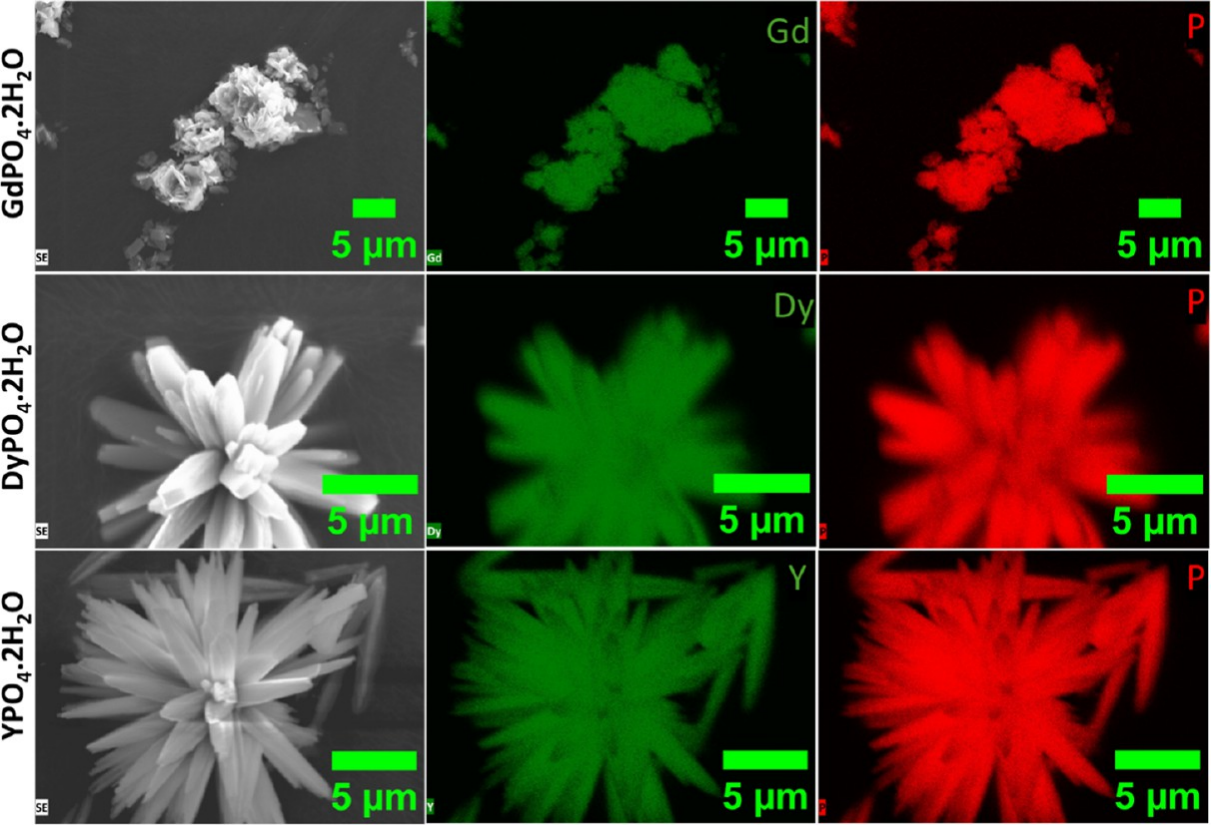
SEM–EDS images of churchite ((Gd/Dy/Y)­PO_4_·2H_2_O) materials.

**Figure 10:**
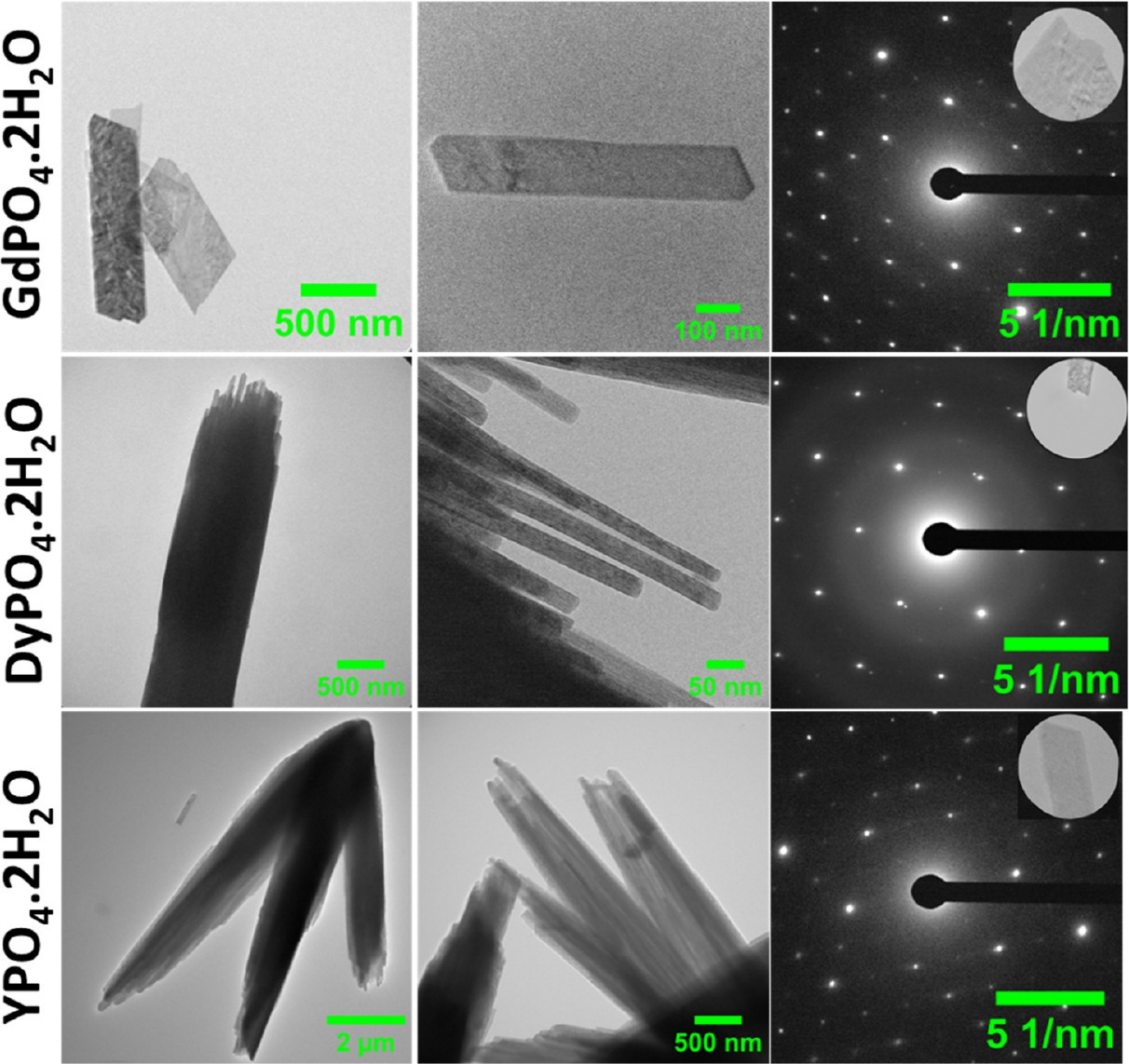
TEM images and SAED patterns of churchite, (Gd/Dy/Y)­PO_4_·2H_2_O, exhibiting the presence of needle-like nanocrystals.

### 3.5 In Situ Ion Irradiation

#### 3.5.1 Xe^2+^ Ion Irradiation of Rhabdophane

The effect of 650 keV Xe^2+^ ion irradiation on the structure of rhabdophane was observed in situ using TEM and representative BF-TEM images and SAED patterns of pristine and ion-irradiated (Sm/Gd/Dy)­PO_4_·H_2_O collected at 298 K are presented in Figure 11. The electron diffraction patterns confirmed the highly crystalline nature of all three materials, exhibiting sharp and bright diffraction spots. The ion-irradiation was performed on an isolated or a group of few nano crystals. At a fluence of ∼2–3 × 10^13^ ions/cm^2^, the decrease in diffraction spot intensities and the concurrent emergence of a diffuse amorphous halo signaled the onset of structural disorder. With increasing ion-fluences, a progressive loss in crystallinity is accompanied by an increase in the amorphous fraction. The ion-fluence at which the material becomes completely amorphous (indicated by the loss of diffraction spots in SAED pattern) is called as the critical amorphization fluence, *F*_c_. No significant trends in the values of *F*_c_ were observed across the series and irrespective of the RE, the as-synthesized rhabdophane materials undergo a radiation-induced amorphization at similar ion fluences (see Figure 11). It is to be noted that the crystalline features that were present in the nano crystals before ion-irradiation were completely lost after ion irradiation (Figure 11).

**Figure 11:**
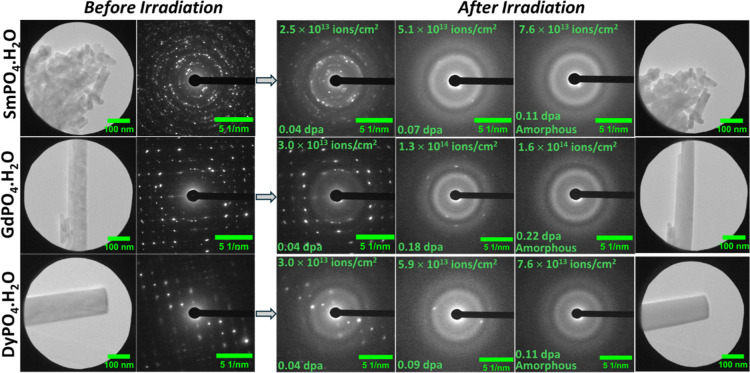
SAED patterns of pristine and Xe^2+^ ion-irradiated rhabdophane, (Sm/Gd/Dy)­PO_4_·H_2_O at 298 K.

The radiation response of (Sm/Gd/Dy)­PO_4_·H_2_O was also investigated as a function of temperature to determine the critical temperature (*T*_c_), defined as the temperature above which amorphization does not occur. The *T*_c_ is a complex parameter highly dependent on intrinsic material characteristics (e.g., chemical composition and crystal structure) and incident ion beam parameters (e.g., ion mass, energy, and flux). An increase in the *F*_c_ was observed for all materials with increasing temperature. This observation is a consequence of the kinetics governing the radiation-induced amorphization process. Amorphization typically occurs when the rate of damage production (e.g., vacancies and interstitials) in a material exceeds the rate of recombination of these point defects. As the irradiation temperature increases, point defect mobility is significantly enhanced, thereby accelerating the recombination of vacancy–interstitial pairs.^73^ Consequently, a higher ion-fluence is required to overcome this thermal barrier to amorphization.

Depending on the RE, the rhabdophane materials underwent radiation-induced amorphization up to 573 K (GdPO_4_·H_2_O) or 673 K (SmPO_4_·H_2_O, DyPO_4_·H_2_O). At 673 K, the GdPO_4_·H_2_O material did not undergo amorphization and remained crystalline up to 1.6 × 10^15^ ions/cm^2^. Similarly, at 723 K, the SmPO_4_·H_2_O and DyPO_4_·H_2_O materials did not undergo transition to an amorphous state and remained crystalline up to 4.2 × 10^15^ ions/cm^2^ and 5.8 × 10^15^ ions/cm^2^, respectively. One method to determine the *T*_c_ of the materials is to define it as the average of the upper temperature limit for which amorphization was observed and the lower temperature limit for which the material remained crystalline and resisted amorphization. Accordingly, using this method, the *T*_c_ of GdPO_4_·H_2_O and (Sm/Dy)­PO_4_·H_2_O is 623 and 698 K, respectively.

The *F*_c_ of (Sm/Gd/Dy)­PO_4_·H_2_O are plotted against the corresponding irradiation temperature (Figure 12) and the *T*_c_ is also determined by fitting this nonlinear curve using eq 1 and by using the experimentally determined temperature limits as mentioned earlier as constraints.^74^

1 $$F_{c}=\frac{F_{c,0}}{1-\exp\left[\left(\frac{E_{a}}{k_{b}}\right)\left(\frac{1}{T_{c}}-\frac{1}{T}\right)\right]}$$

**Figure 12:**
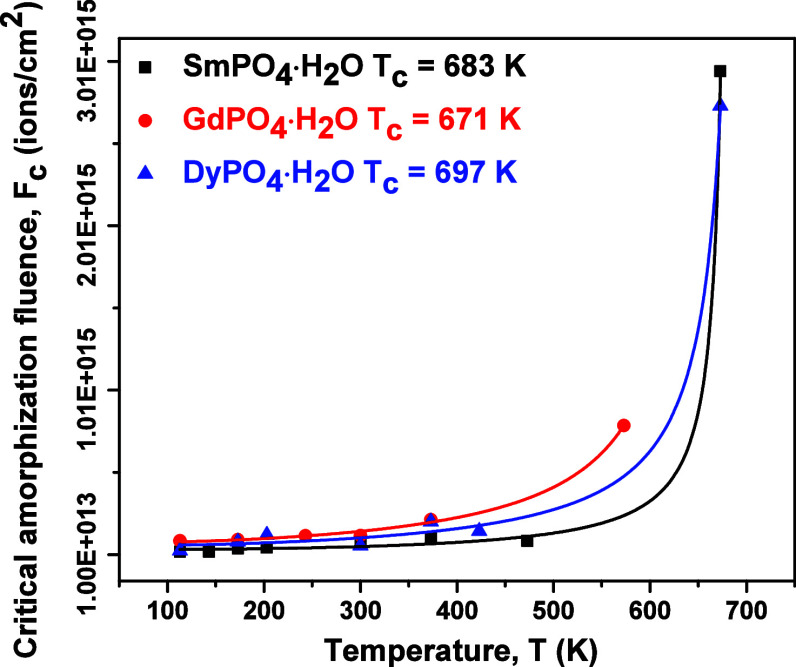
Plot of critical amorphization fluence (*F*_c_) versus irradiation temperature (*T*) for rhabdophane materials. The data points were fitted using eq 1.

In this equation, *F*_c_ and *F*_c,0_ represents the critical amorphization fluence at a given irradiation temperature *T* and 0 K, respectively. The *F*_c,0_ is obtained by extrapolating the curve to *T* = 0 K. *E*_a_ represents the activation energy for the annealing or recrystallization of amorphous domains, *k*_b_ is the Boltzmann’s constant. During the fitting procedure, the following parameters were varied: *F*_c,0_, *E*_a_, and *T*_c_. The fit parameters of REPO_4_·H_2_O are presented in Table 3 along with the *T*_c_ values obtained from the experiment. No significant differences in *T*_c_ were observed as a function of the RE; however, the *T*_c_ of GdPO_4_·H_2_O is slightly lower than those of SmPO_4_·H_2_O and DyPO_4_·H_2_O.

**Table 3:** *F*_c_ Versus *T* Curve–Fit Parameters for Rhabdophane, (Sm/Gd/Dy)­PO_4_·H_2_O

rhabdophane	*T*_c_ (K) (curve-fit)	*T*_c_ (K) (experiment)	*F*_c,0_ (ions/cm^2^)	*E*_a_ (eV)
SmPO_4_·H_2_O	683 ± 4	698	4.2 (1.4) × 10^13^	0.06 (0.03)
GdPO_4_·H_2_O	674 ± 18	623	8.5 (1.7) × 10^13^	0.04 (0.01)
DyPO_4_·H_2_O	697 ± 11	698	6.6 (4.1) × 10^13^	0.04 (0.03)

#### 3.5.2 Xe^2+^ Ion Irradiation of Churchite

The BF-TEM images and SAED patterns of pristine and ion-irradiated churchite materials at 298 K are presented in Figure 13. Isolated or a bunch of electron-transparent needle-like nanocrystals were chosen for ion irradiation. The pristine materials are highly crystalline, as indicated by the presence of sharp diffraction spots in their SAED patterns. Upon irradiation, the materials become increasingly disordered as revealed by the decrease in the diffraction spot intensities and the appearance of an amorphous halo. With increasing ion fluence, the materials become completely amorphous, and the needle-like nanocrystals lose all their crystalline features that were present preirradiation. The churchite materials have similar *F*_c_ values; however, the *F*_c_ (and dpa) values of churchite were found to be lower than those of rhabdophane (cf. Figures 11 and 13). This observation suggests that the churchite materials are highly radiation sensitive and are more susceptible to amorphization than rhabdophane. These differences in *F*_c_ between churchite and rhabdophane can be likely attributed to their different crystal structures. The churchite materials adopt a layered structure in which the layers are held together by interlayer H bonding. On the other hand, rhabdophane adopts a framework structure in which the water molecules occupy the channels. It is well-known that churchite materials have lower thermal stability than the rhabdophane and that upon complete removal of water molecules, the layered structure collapses and transforms to a more stable xenotime structure.^57^ However, the rhabdophane material maintains its framework structure even after the removal of water, albeit up to a certain temperature beyond which it also transforms to a more stable monazite structure.^56^ Based on these structural differences, the relatively lower thermal stability of churchite suggests that it is more susceptible to amorphization. Consequently, Xe^2+^ ions are expected to efficiently induce amorphization more easily by disrupting the interlayer H-bonding, resulting in a collapse of the layered structure. The relatively lower *F*_c_ of churchite could also be explained using the topological model proposed by Hobbs.^75^ According to this model, materials are considered less susceptible to amorphization if they possess a higher connectivity of the cation polyhedra in their crystal structures.^75^ In the churchite, the connectivity of the REO_8_ polyhedra is interrupted by the presence of interlayer water molecules, and hence, their average connectivity is lower than those found in the framework structure of rhabdophane. Thus, churchite has a lower *F*_c_ value in comparison to the rhabdophane structure.

**Figure 13:**
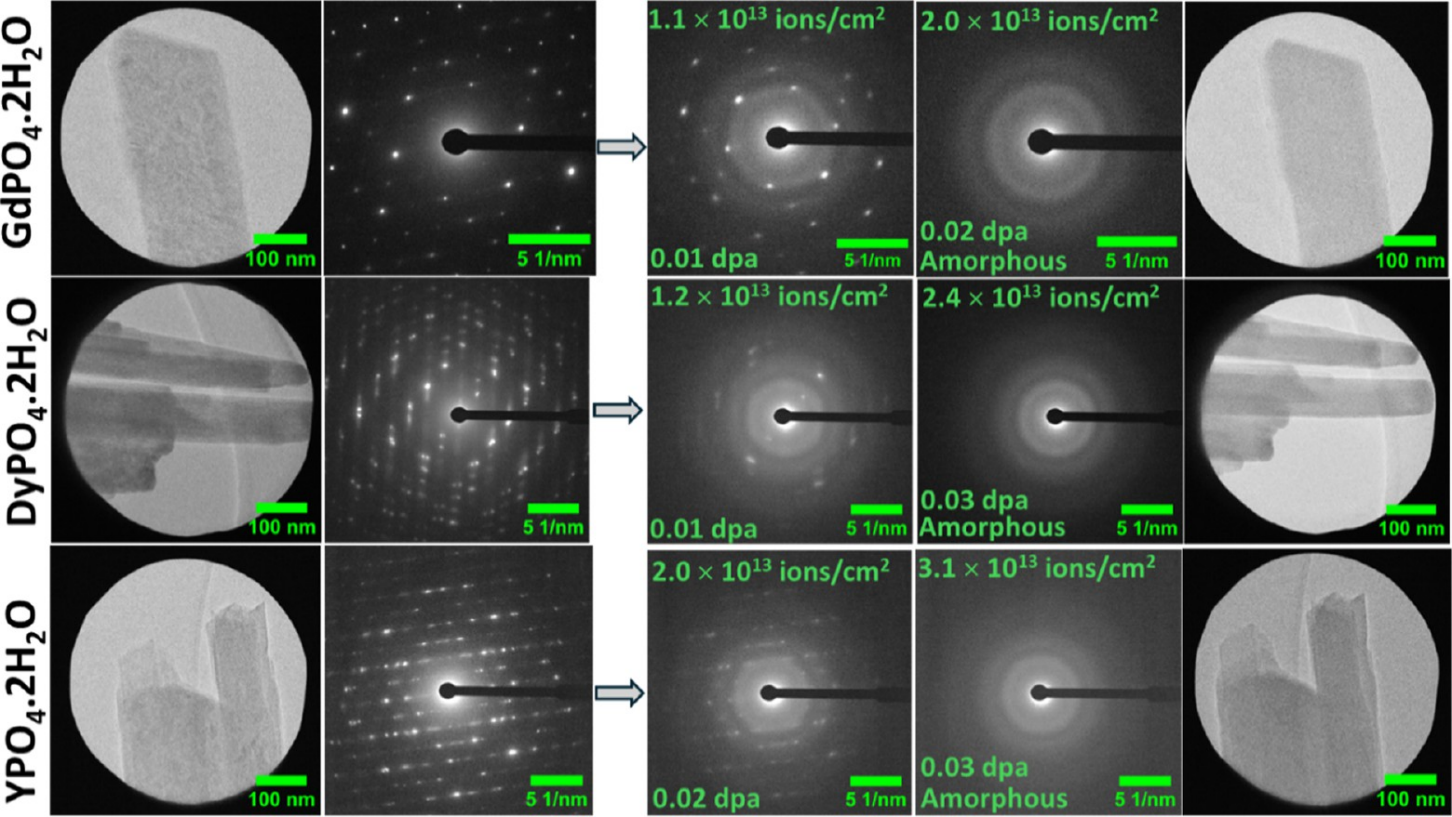
SAED patterns of pristine and Xe^2+^ ion-irradiated churchite, (Gd/Dy/Y)­PO_4_·2H_2_O at 298 K.

To determine the *T*_c_ of (Gd/Dy/Y)­PO_4_·2H_2_O, the *F*_c_ was measured across a range of irradiation temperatures (*T*). However, as discussed earlier, the churchite materials are metastable phases with low thermal stability; previous studies by the authors have demonstrated that churchite retains its structure only up to 473 K, transforming into the thermodynamically stable xenotime phase at temperatures >473 K.^57^ Hence, in this study, the *F*_c_ was measured up to 473 K. Unlike the rhabdophane materials, the churchite material did not exhibit a significant increase in *F*_c_ with increasing temperature. Amorphization was observed at all temperatures up to 473 K, indicating that the temperature required for defect recombination likely exceeds the thermal stability limit of the material. Given the competing phase transition of churchite to xenotime at *T* > 473 K, the *T*_c_ cannot be determined experimentally for the churchite phase.

#### 3.5.3 Xe^2+^ Ion Irradiation of Monazite (SmPO_4_) and Xenotime (DyPO_4_)

To benchmark the radiation response of the hydrous metastable phases against their primary stable anhydrous counterparts, ion irradiation experiments were conducted on anhydrous materials, monazite (SmPO_4_) and xenotime (DyPO_4_). The monazite and xenotime materials were synthesized by annealing the Sm-rhabdophane and Dy-rhabdophane to 1000 °C (1273 K), respectively. The BF-TEM and SAED patterns of pristine and ion-irradiated (Sm/Dy)­PO_4_ at 298 K are presented in Figure 14. The (Sm/Dy)­PO_4_ has a higher *F*_c_ than those of their hydrous counterparts, rhabdophane (Sm/Dy)­PO_4_·H_2_O and churchite (DyPO_4_·2H_2_O). Unlike the hydrous materials, the REPO_4_ (RE = Sm, Dy) are thermodynamically stable phases with a higher connectivity of the REO_*n*_ (*n* = 8 or 9) cationic polyhedra and hence, higher ion fluence is required to induce a structural transformation to amorphous state in these materials.

**Figure 14:**
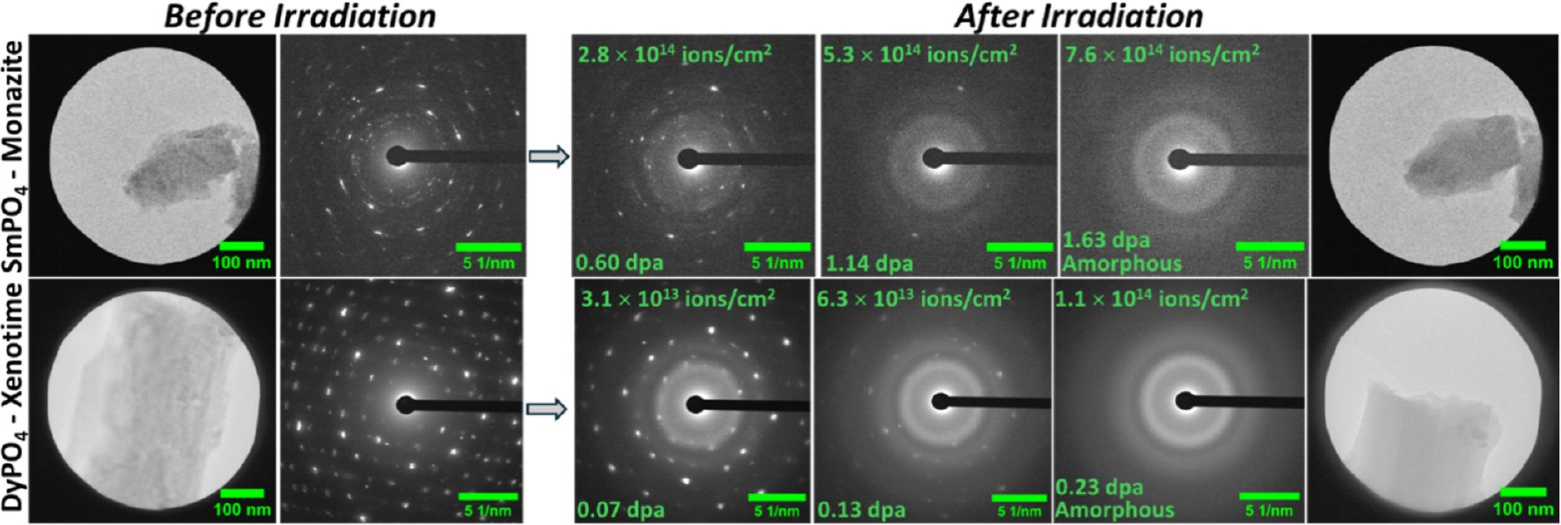
SAED patterns of pristine and Xe^2+^ ion-irradiated monazite (SmPO_4_) and xenotime (DyPO_4_) materials at 298 K.

Between the REPO_4_, the monazite-type SmPO_4_ has an *F*_c_ higher than that of the xenotime-type DyPO_4_. This observation is consistent with the studies reported in the literature and could be explained using their respective crystal structures.^16,19^ The SmO_9_ polyhedra in the monazite structure are distorted, whereas the DyO_8_ polyhedra in the xenotime structure are more symmetrical. As a result, it can be inferred that the monazite structure offers greater flexibility and tolerance toward accommodating radiation-induced structural defects within the lattice. In addition, according to the Hobbs model, the monazite structure which has a higher connectivity of the REO_9_ polyhedra is considered more radiation-tolerant in comparison to the xenotime structure which has a relatively lower connectivity of the REO_8_ polyhedra.^13,19^

The *T*_c_ of the (Sm/Dy)­PO_4_ was also determined by investigating their radiation response as a function of temperature. The general trend is that the *F*_c_ increases with increasing temperature. In monazite (SmPO_4_) and xenotime (DyPO_4_), amorphization was observed up to temperatures of 523 and 673 K, respectively. The monazite material did not undergo amorphization at 573 K and remained crystalline. In the case of xenotime, the material resisted further amorphization at 723 K. The *T*_c_ of the materials was determined from the average of the highest temperature at which amorphization was observed and the lowest temperature at which amorphization was not observed. Accordingly, the *T*_c_ of SmPO_4_ and DyPO_4_ were determined to be 548 and 698 K, respectively. The *T*_c_ of monazite (SmPO_4_) are significantly lower in comparison to the *T*_c_ of rhabdophane (SmPO_4_·H_2_O) material. This observation reflects the ability of anhydrous REPO_4_ to efficiently recover from radiation-induced structural damage by enabling the recombination of defects such as vacancies and interstitials at relatively lower temperatures. However, no trends were observed between the *T*_c_ values of xenotime (DyPO_4_) and Dy-rhabdophane (DyPO_4_·H_2_O) and were found to be similar. The *F*_c_, Amorphization dpa, and *T*_c_ values of anhydrous and hydrous rare-earth phosphates are summarized in Table 4. It can be deduced from Table 4 that the churchite structure has the lowest *F*_c_ (and lowest dpa) and is the least radiation-tolerant. Using the values of *F*_c_, radiation stability order can be constructed as follows: churchite < rhabdophane < xenotime < monazite. Notably, this radiation stability order is identical to the thermodynamic stability order of these crystal structures determined from high temperature calorimetry experiments.^40^

**Table 4:** *F*_c_ and *T*_c_ of Hydrous and Anhydrous Rare-Earth Phosphates

composition	*F*_c_ (ions/cm^2^)	amorphization dpa	*T*_c_—experimental (K)
**Churchite**			
GdPO_4_·2H_2_O	2.0 × 10^13^	0.02 dpa	-
DyPO_4_·2H_2_O	2.4 × 10^13^	0.03 dpa	-
YPO_4_·2H_2_O	3.1 × 10^13^	0.03 dpa	-
**Rhabdophane**			
SmPO_4_·H_2_O	7.6 × 10^13^	0.11 dpa	698
GdPO_4_·H_2_O	1.6 × 10^14^	0.22 dpa	623
DyPO_4_·H_2_O	7.6 × 10^13^	0.11 dpa	698
**Xenotime**			
DyPO_4_	1.1 × 10^14^	0.23 dpa	698
**Monazite**			
SmPO_4_	7.6 × 10^14^	1.63 dpa	548

Between monazite and xenotime, the *T*_c_ of the monazite is significantly lower than xenotime and indicates the monazite structure’s capacity to efficiently anneal radiation-induced structural defects at lower temperatures. The observed differences are directly linked to their variations in their crystal structure and their ability to accommodate and anneal structural defects. The *T*_c_ of the monazite and xenotime were also determined by fitting the *F*_c_ versus temperature curves using eq 1 (Figure 15). The values of the fit parameters (*T*_c_, *E*_a_, *F*_c,0_) are presented in Table 5. The *F*_c_ versus *T* plots of monazite and xenotime are also compared with rhabdophane and are presented in Figure 15. Clearly, the monazite material has a significantly lower *T*_c_ than rhabdophane.

**Figure 15:**
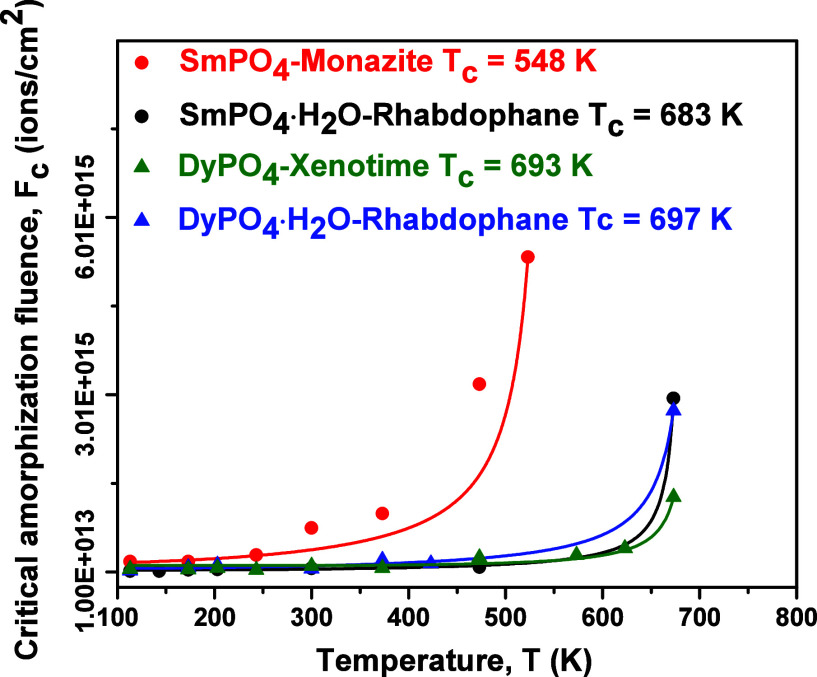
Plot of critical amorphization fluence (*F*_c_) versus irradiation temperature (*T*) for monazite, xenotime, and rhabdophane. The data points were fitted using eq 1.

**Table 5:** *F*_c_ Versus *T* Curve-Fit Parameters of Monazite, Xenotime, and Rhabdophane

composition	*T*_c_ (K) (curve-fit)	*T*_c_ (K) (experiment)	*F*_c,0_ (ions/cm^2^)	*E*_a_ (eV)
SmPO_4_ (monazite)	548 ± 7	548	1.6 (4.1) × 10^14^	0.03 (0.01)
DyPO_4_ (xenotime)	693 ± 7	698	1.1 (3.6) × 10^14^	0.2 (0.1)
SmPO_4_·H_2_O (rhabdophane)	683 ± 4	698	4.2 (1.4) × 10^13^	0.06 (0.03)
DyPO_4_·H_2_O (rhabdophane)	697 ± 11	698	6.6 (4.1) × 10^13^	0.04 (0.03)

## 4 Implications

The susceptibility of secondary actinide host phases to radiation-induced amorphization has significant implications for the fate of actinides immobilized within these phases in natural and geological repository environments.^48,76,77^ This is particularly relevant at legacy nuclear waste sites such as Hanford (USA), wherein evidence of Pu incorporation in the rhabdophane structure is observed in soil sediments beneath disposal trenches.^48^ Given that rhabdophane-type structures are shown in this study to be susceptible to radiation damage, the resulting loss of crystallinity may diminish its chemical durability, potentially facilitating Pu release and subsequent migration. Similarly, if monazite or xenotime waste forms are utilized for HLW immobilization, then aqueous alteration in a geological repository could lead to the formation of rhabdophane and churchite phases. In this scenario, actinides partition into these secondary phases, which are then subjected to radiation damage and could result in the release of actinides into the environment.

## 5 Conclusions

Current performance assessments of nuclear waste forms (e.g., monazite and xenotime) predominantly focus on the primary host matrix, while the radiation stability of secondary alteration phases (e.g., rhabdophane and churchite)—which may form under repository conditions—is often overlooked. In this study, the α-recoil induced structural changes in secondary hydrous rare earth phosphates (rhabdophane—REPO_4_·H_2_O; churchite—RE′PO_4_·2H_2_O) were investigated for the first time via in situ ion irradiation. Both rhabdophane and churchite undergo radiation-induced amorphization; however, it was observed that the rhabdophane materials have a relatively higher critical amorphization fluence (*F*_c_) in comparison to the churchite materials.

The radiation response of these materials was also monitored as a function of temperature to evaluate the critical amorphization temperature (*T*_c_). Depending on RE, the rhabdophane materials have a *T*_c_ in the range of 623 to 698 K. The rhabdophane materials are stable up to ∼873 K and transform to a monazite (REPO_4_; RE = Sm, Gd) or xenotime (RE′PO_4_; RE′ = Dy) phase beyond this temperature. The *T*_c_ of rhabdophane determined in this study is well below its thermal stability limit (∼873 K). The *T*_c_ of churchite materials could not be established because they become amorphous at all temperatures up to their thermal stability limit (∼473 K). At temperatures above 473 K, the churchite materials transform to a xenotime (RE′PO_4_; RE′ = Gd, Dy, Y) phase. The radiation tolerance of the metastable hydrous rare-earth phosphates was compared with their thermodynamically stable anhydrous counterparts, monazite (SmPO_4_) and xenotime (DyPO_4_). It was found that the rhabdophane and churchite materials have a lower *F*_c_ than the monazite and xenotime materials. Similarly, the *T*_c_ of rhabdophane (REPO_4_·H_2_O = 698 K; RE = Sm, Dy) materials was also higher than monazite (SmPO_4_—548 K).

This study has shed light on the radiation stability of the hydrous rare-earth phosphates, and the results show that the rhabdophane and churchite materials, which could potentially form because of chemical alteration of monazite and xenotime waste forms, are more susceptible to α-recoil induced structural damage than their anhydrous counterparts. This finding could have implications on the chemical durability and consequently, the actinide retention capability of these secondary phases. It is also proposed that the local temperatures (∼320–400 K) within the geological repository environment are anticipated to mitigate radiation damage in these secondary phases, either by suppressing amorphization during irradiation or by promoting the thermal recovery of damaged zones. Building on the present findings, subsequent experimental studies are necessary to establish the correlation between structural damage and leach resistance in these secondary phases, thereby enabling a comprehensive evaluation of their actinide retention capacity under repository-relevant conditions.

## Supplementary Material



## Notes

Lattice constants of monazite and xenotime, simulated XRD patterns of rhabdophane-type CePO_4_·*n*H_2_O material, Rietveld refined powder XRD patterns from rhabdophane, powder XRD patterns of rhabdophane annealed to 600 °C, Rietveld refined powder XRD patterns from churchite, powder XRD pattern from monazite, powder XRD pattern from xenotime, Rietveld refined powder XRD patterns from monazite, Rietveld refined powder XRD patterns from xenotime, additional SEM images of Dy-rhabdophane indicating the prismatic facets of the nanorods, EDS spectra of rhabdophane, and EDS spectra of churchite (PDF)
