# Supplementary material for: In Situ Observation of Radiation Damage in Rhabdophane- and Churchite-Type Rare-Earth Phosphate Nanocrystals
Source: Inorg Chem. 2026 May 25;65(22):12509–23. doi: 10.1021/acs.inorgchem.6c01299 (PMC13250987; doi:10.1021/acs.inorgchem.6c01299)

Supporting information for

In situ observation of radiation damage in rhabdophane and churchite-type rare-earth phosphate nanocrystals

Mohamed Ruwaid Rafiuddin[1*], Anamul Haq Mir[2], Yingjie Zhang[1]

[1]Australian Nuclear Science and Technology Organisation, Locked Bag 2001, Kirrawee DC, NSW 2232, Australia

[2]Department of Earth Sciences, University of Cambridge, Downing Site, CB2 3EQ, United Kingdom

*Corresponding Author

E-mail address: rafiuddr@ansto.gov.au

Table S1 Lattice constants of monazite ($SmPO_4$) and xenotime ($DyPO_4$)

Material	a (Å)	b (Å)	c (Å)	β (°)	Unit cell volume ($Å^3$)
Monazite – $SmPO_4$	6.69099 (7)	6.89355 (7)	6.37161 (6)	103.8606 (7)	285.3
Xenotime – $DyPO_4$	6.9168 (1)	-	6.0504 (2)	-	289.5

Figure S1 Simulated powder XRD patterns from rhabdophane-type $CePO_4 \cdot H_2O$ with $P3_121$ and $P6_222$ space groups

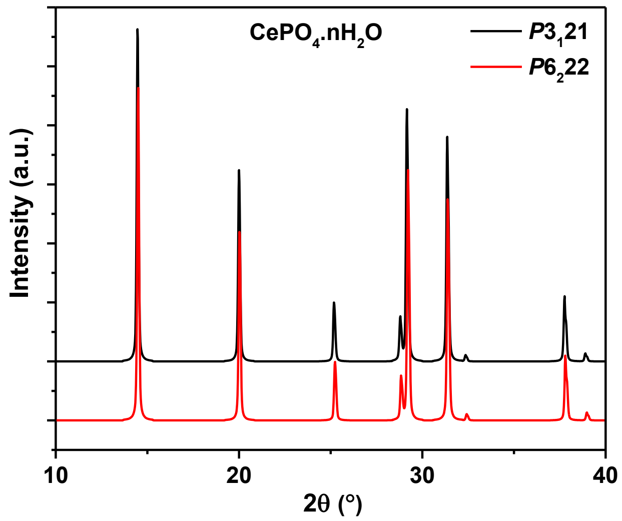

Figure S2 Rietveld refined powder XRD patterns from rhabdophane (Sm/Gd/Dy)$PO_4 \cdot H_2O$

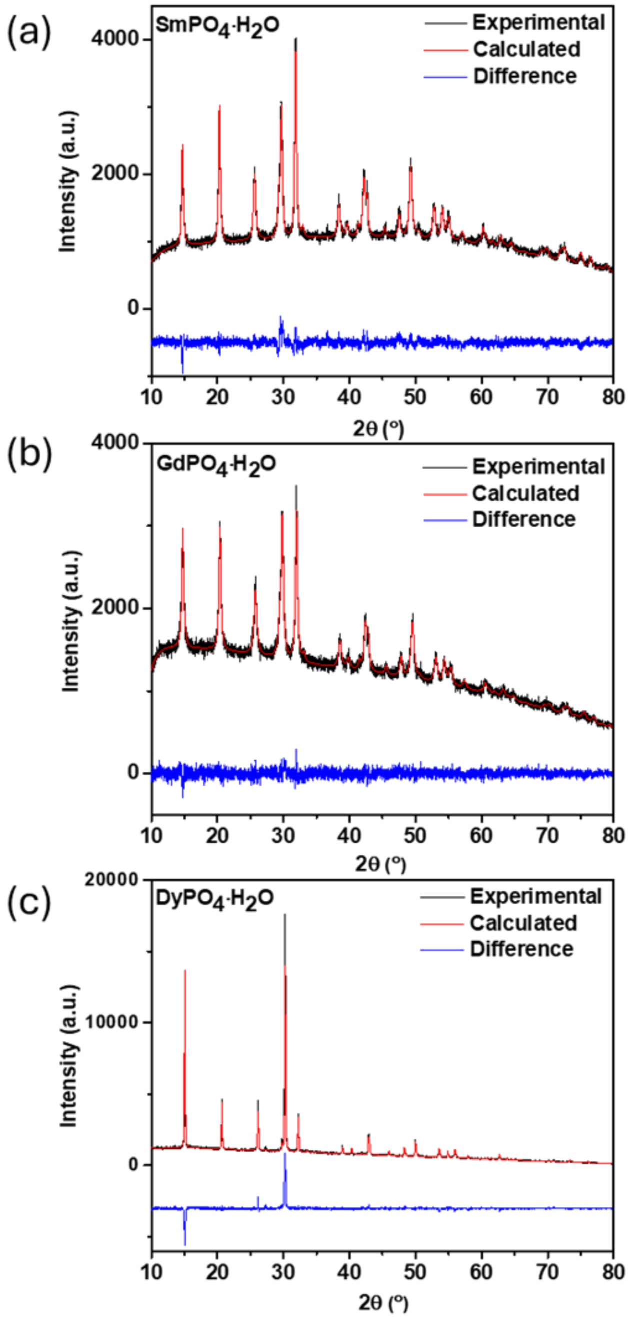

Figure S3 Powder XRD patterns of rhabdophane annealed to 600°C

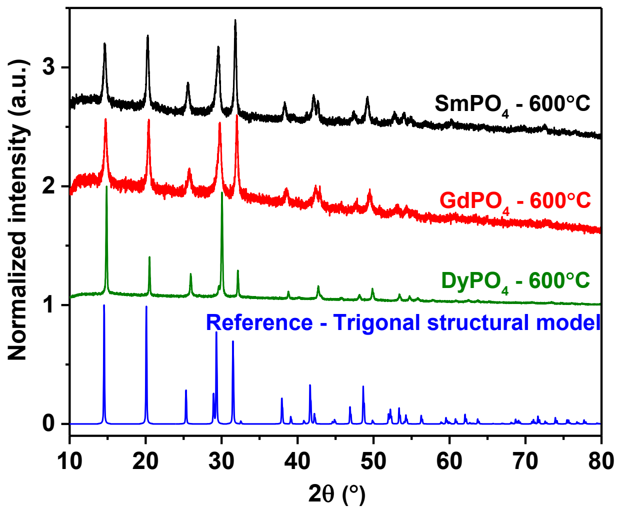

Figure S4 Rietveld refined powder XRD patterns from churchite (Gd/Dy/Y)$PO_4·2H_2O$

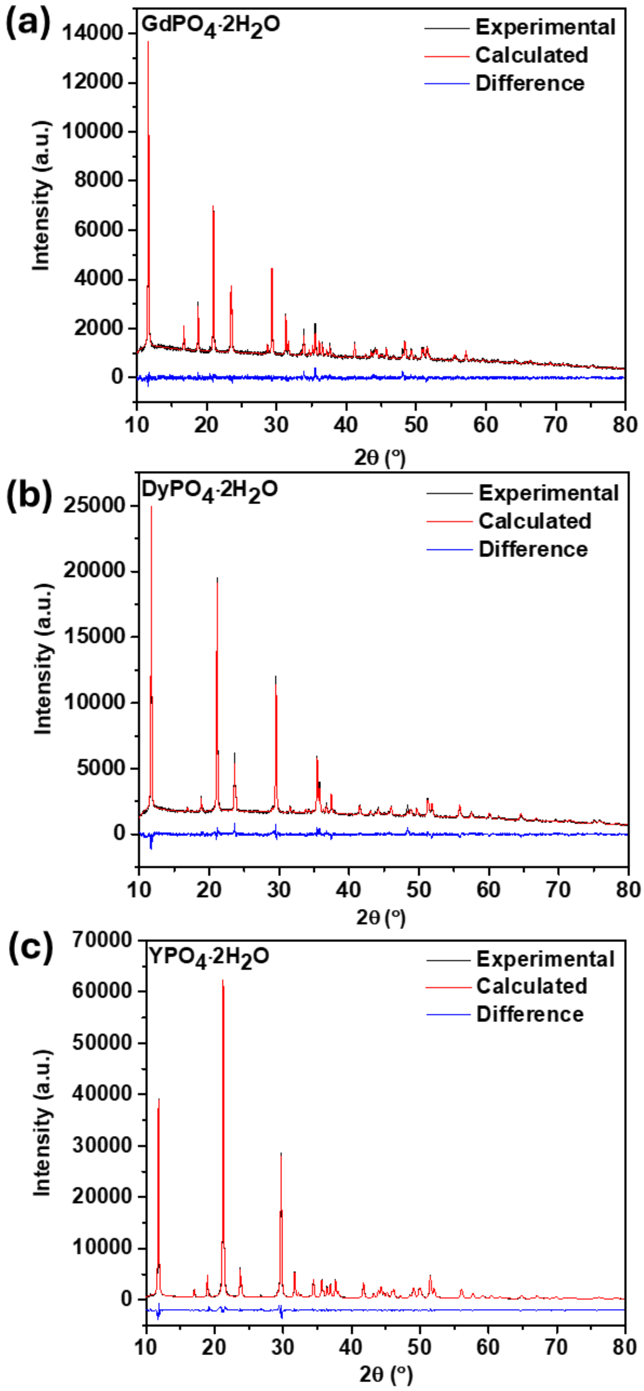

Figure S5 Powder XRD patterns from $SmPO_4$ – 1000°C

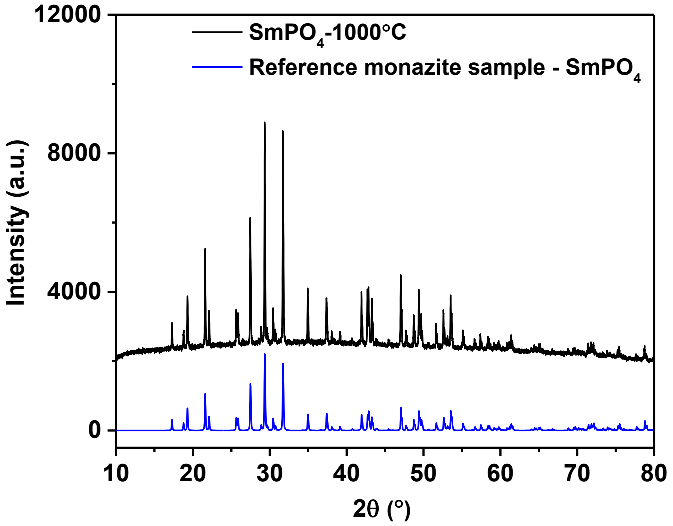

Figure S6 Powder XRD patterns from $DyPO_4$-– 1000°C

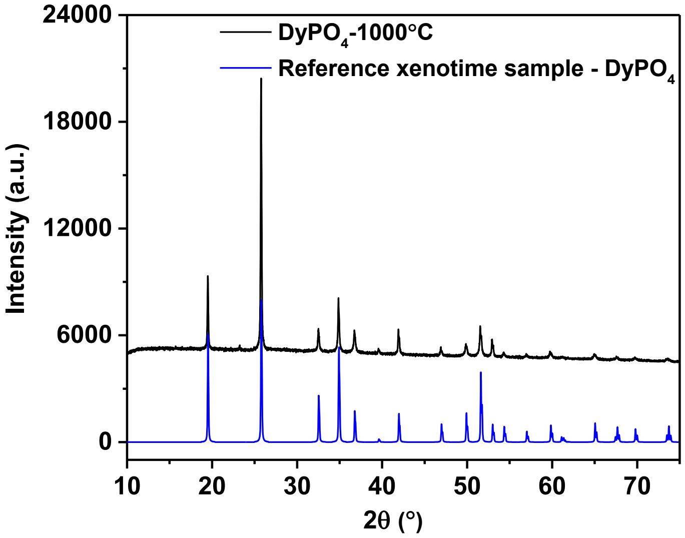

Figure S7 Rietveld refined powder XRD patterns from Monazite, $SmPO_4$

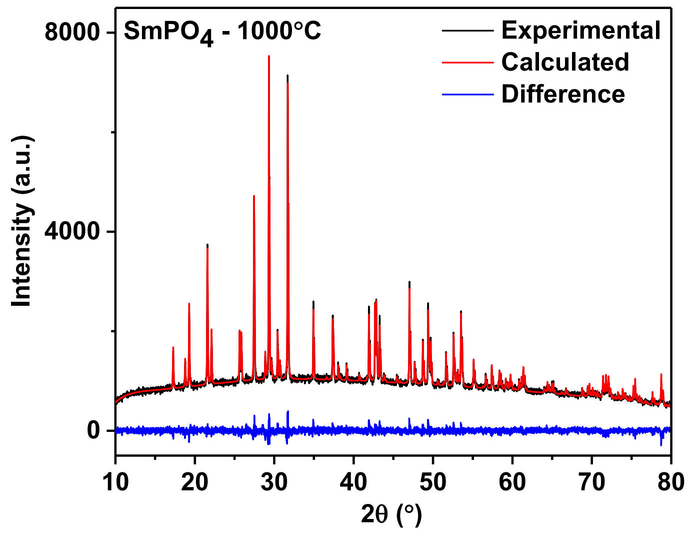

Figure S8 Rietveld refined powder XRD patterns from Xenotime, $DyPO_4$

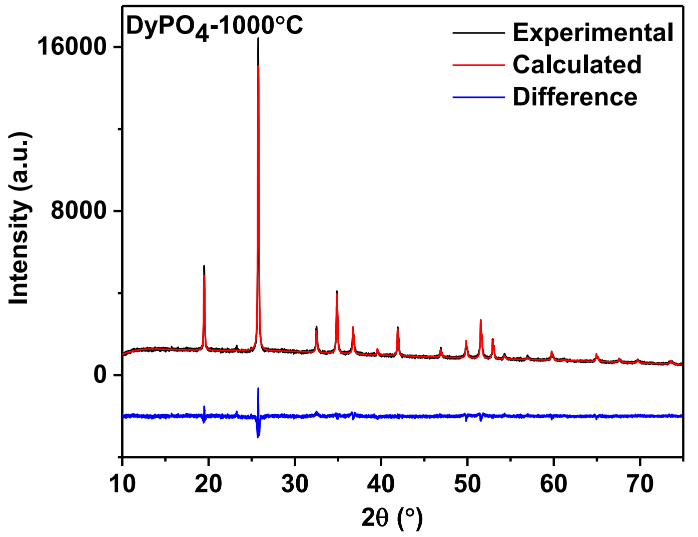

Figure S9 High resolution SEM images of rhabdophane, $DyPO_4 \cdot H_2O$. Arrows indicate the prismatic facets of the nanorods. SEM images were obtained using a 10 kV accelerating voltage from a Zeiss Ultra Plus SEM (Carl Zeiss NTS GmbH).

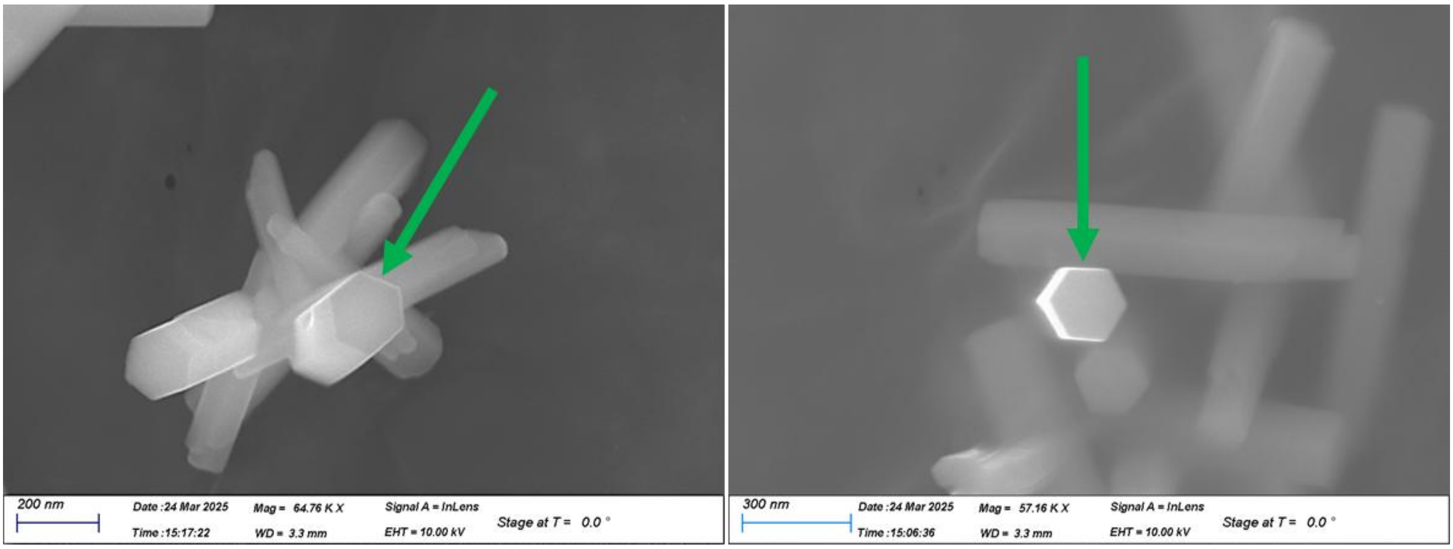

Figure S10 EDS spectra of rhabdophane (Sm/Gd/Dy)$PO_4 \cdot H_2O$

(a) $SmPO_4 \cdot H_2O$

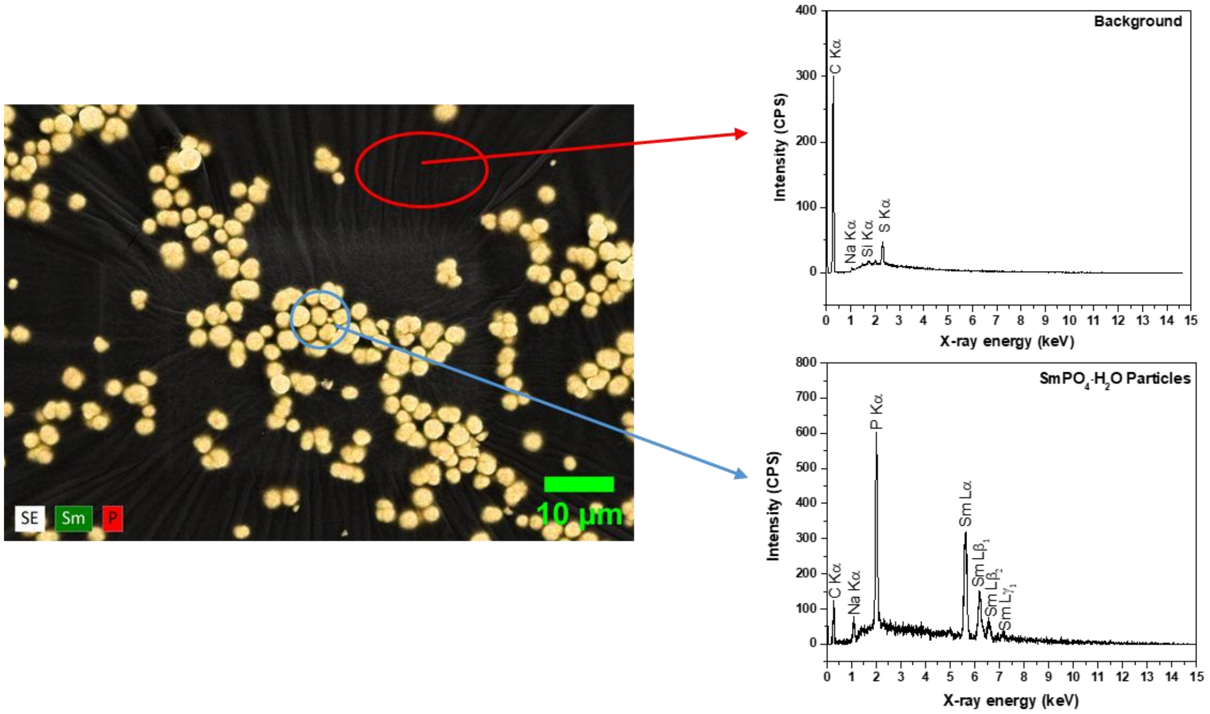

(b) $GdPO_4 \cdot H_2O$

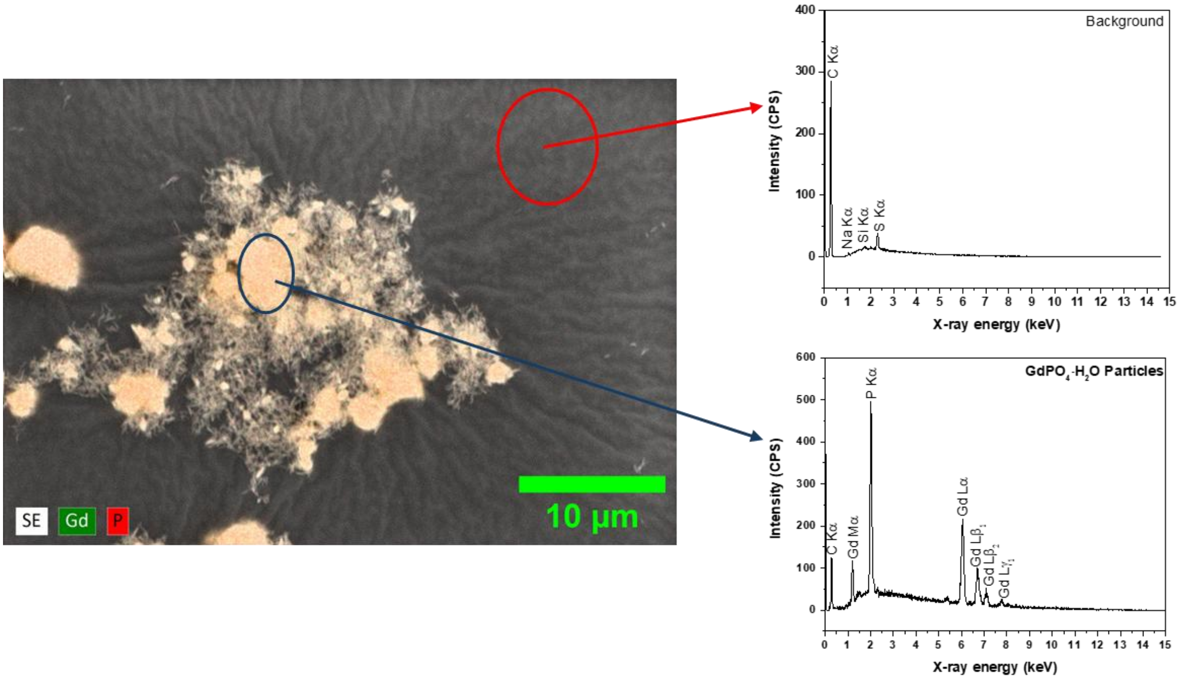

(c) $DyPO_4 \cdot H_2O$

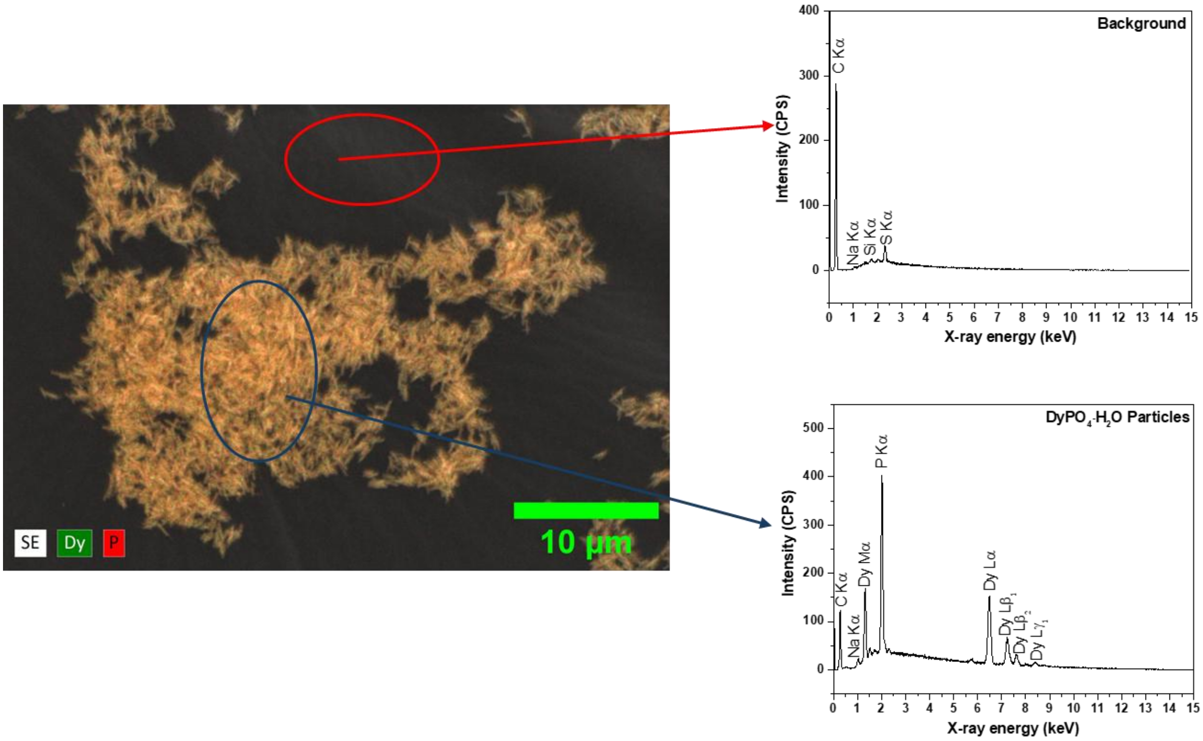

Figure S11 EDS spectra of Churchite (Gd/Dy/Y)$PO_4 \cdot 2H_2O$

(a) $GdPO_4 \cdot 2H_2O$

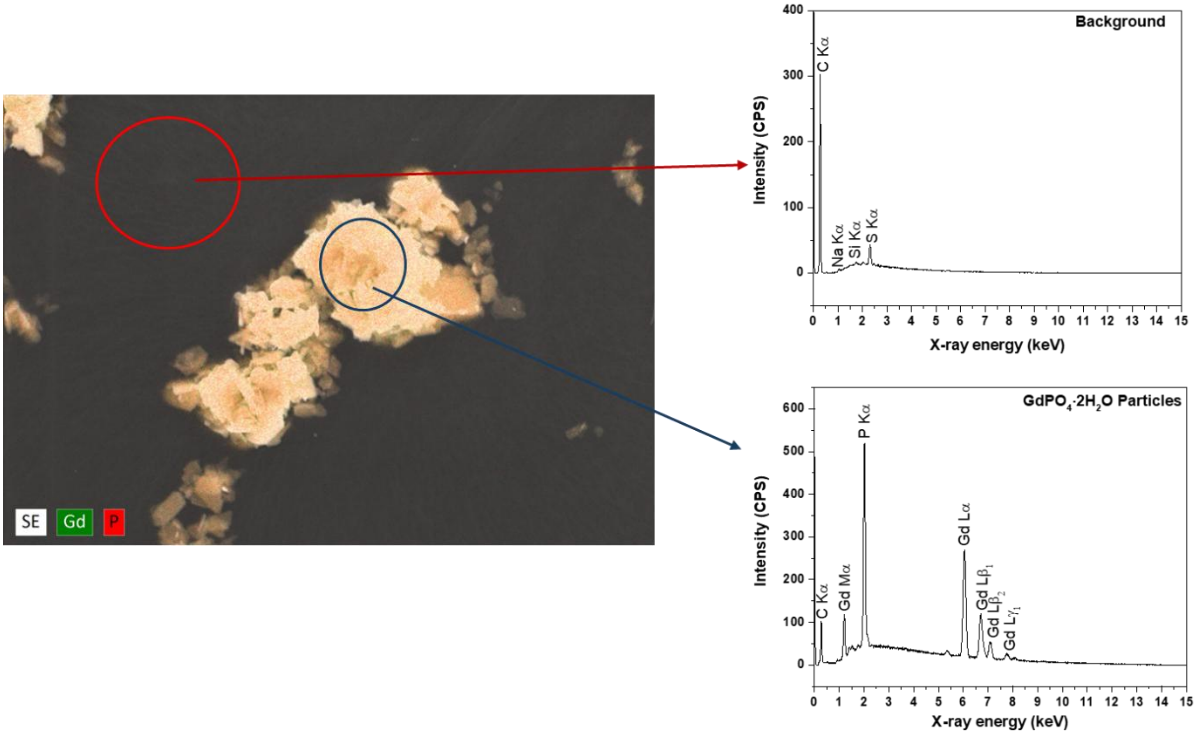

(b) $DyPO_4 \cdot 2H_2O$

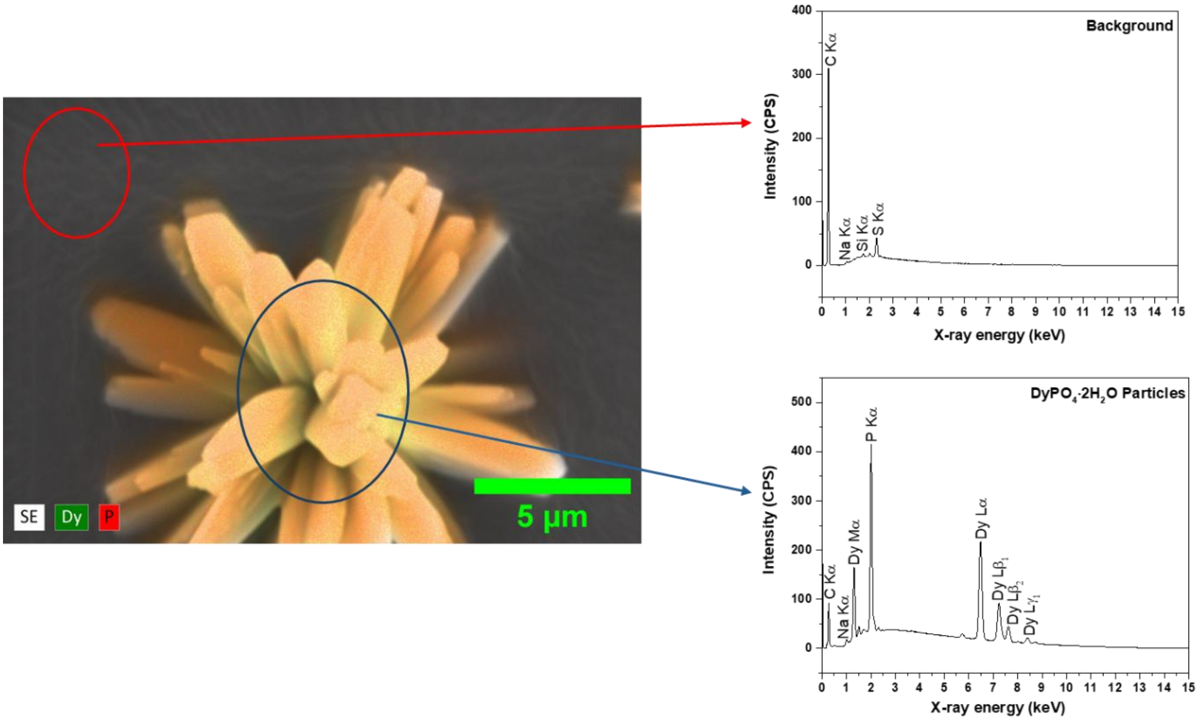

(c) $YPO_4{\cdot}2H_2O$

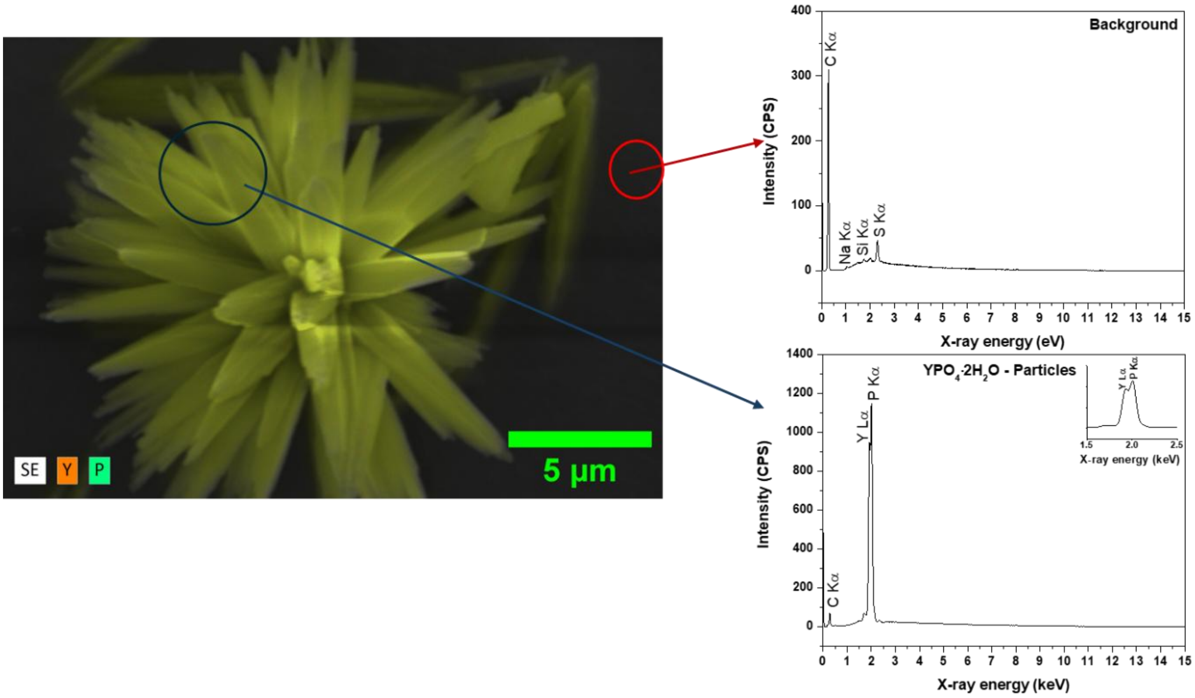

Supplement: Supplementary file 1 [file ic6c01299_si_001.pdf]
